# Biocontrol agents *promote* growth of potato pathogens, depending on environmental conditions

**DOI:** 10.1111/1751-7915.12349

**Published:** 2016-02-16

**Authors:** Jonathan A. Cray, Mairéad C. Connor, Andrew Stevenson, Jonathan D. R. Houghton, Drauzio E. N. Rangel, Louise R. Cooke, John E. Hallsworth

**Affiliations:** ^1^ Institute for Global Food SecuritySchool of Biological SciencesMBCQueen's University BelfastBelfastBT9 7BLNorthern Ireland; ^2^ Instituto de Patologia Tropical e Saúde PúblicaUniversidade Federal de GoiásGoiâniaGO74605‐050Brazil; ^3^ Agri‐Food & Biosciences Institute (AFBI)Newforge LaneBelfastBT9 5PXNorthern Ireland

## Abstract

There is a pressing need to understand and optimize biological control so as to avoid over‐reliance on the synthetic chemical pesticides that can damage environmental and human health. This study focused on interactions between a novel biocontrol‐strain, *Bacillus* sp. JC12GB43, and potato‐pathogenic *Phytophthora* and *Fusarium* species. In assays carried out *in vitro* and on the potato tuber, the bacterium was capable of near‐complete inhibition of pathogens. This *Bacillus* was sufficiently xerotolerant (water activity limit for growth = 0.928) to out‐perform *Phytophthora infestans* (~0.960) and challenge *Fusarium coeruleum* (~0.847) and *Fusarium sambucinum* (~0.860) towards the lower limits of their growth windows. Under some conditions, however, strain JC12GB43 stimulated proliferation of the pathogens: for instance, *Fusarium coeruleum* growth‐rate was increased under chaotropic conditions in vitro (132 mM urea) by >100% and on tubers (2‐M glycerol) by up to 570%. Culture‐based assays involving macromolecule‐stabilizing (kosmotropic) compatible solutes provided proof‐of‐principle that the *Bacillus* may provide kosmotropic metabolites to the plant pathogen under conditions that destabilize macromolecular systems of the fungal cell. Whilst unprecedented, this finding is consistent with earlier reports that fungi can utilize metabolites derived from bacterial cells. Unless the antimicrobial activities of candidate biocontrol strains are assayed over a full range of field‐relevant parameters, biocontrol agents may promote plant pathogen infections and thereby reduce crop yields. These findings indicate that biocontrol activity, therefore, ought to be regarded as a mode‐of‐behaviour (dependent on prevailing conditions) rather than an inherent property of a bacterial strain.

## Introduction

Potatoes are the fifth most important crop in the world (after wheat, corn, rice and sugar cane) and are an essential food source for 1.3 billion people (Jones, 2013). The modern potato, *Solanum tuberosum*, was domesticated between 7 000 and 10 000 years ago and remains susceptible to attack from microbial pathogens (Spooner *et al*., 2005). This causes losses of potato tuber yields that typically range from 15% to 25%, depending on geographical region and climatic conditions (Oerke, 2006). European‐Union legislation prohibits use of pesticides that are highly mutagenic, carcinogenic and/or teratogenic; and those which are environmentally persistent, bioaccumulate and/or exhibit either acute or chronic toxicity in animal systems (Jess *et al*., 2014). Even for approved pesticides, legislation also limits the quantity of active ingredient that may be applied (Jess *et al*., 2014). Although modifications of the plant genome have enhanced pathogen control for some plant species, *S. tuberosum* is a highly heterozygous tetraploid and the progress of breeding programmes has been accordingly slow (Barrell *et al*., 2013). There is, therefore, a need for effective biological control strategies to protect both potato crops and stored potato tubers. Although biological control alone may not be sufficiently effective (biologically or financially) to displace synthetic pesticides, integrated systems of pathogen control help to safeguard environmental and human health, and can reduce selection pressures for pesticide‐resistant pathogen strains.

For *Fusarium* species that cause dry rot of potato tubers or *Phytophthora infestans* that causes late blight in potato plants, there are no known biocontrol agents as efficacious as synthetic chemical pesticides (Stephan *et al*., 2005; Gachango *et al*., 2012). Furthermore, there is little information on interactions between such potato pathogens and other microbes encountered in the field (or on the stored tuber) which may have potential as biocontrol agents (Schisler *et al*., 1995; Slininger *et al*., 2007; Clermont *et al*., 2011). Many plant surfaces, including those within the phyllosphere, represent fertile habitats for microorganisms (Cray *et al*., 2013a; Lievens *et al*., 2015). Indeed, when there is sufficient water, the phyllosphere can present nutrient‐rich habitats that are open to inhabitation by a significant diversity of microbial life and characterized, therefore, by intense competition, known as ‘open habitats’ (Cray *et al*., 2013a). The dynamics of microbial ecology within these open habitats can enable the emergence of (an) ecologically aggressive microbe(s) that can potentially be exploited for biological control of plant pathogens (Cray *et al*., 2013a). This said, the ability to dominate the microbial community is also known to be habitat‐specific, and can depend intimately on the prevailing conditions (Cray *et al*., 2013a). In the context of biological control, the implications of this finding have not yet been determined.

We hypothesized that, under some circumstances, biocontrol agents may actually *promote* the growth of plant pathogens. Accordingly, the overarching goal of the current study was to obtain both novel and established biocontrol agents, with inhibitory activities against potato pathogens, and characterize the ecology of biocontrol agent:pathogen interactions. The specific aims were to: (1) elaborate a sampling rationale to obtain, and culture, novel bacterial (and other microbial) strains likely to have biocontrol potential against potato pathogens, (2) determine the outcomes of interactions between these microbes, and established biocontrol agents, and pathogenic isolates of *Fusarium coeruleum*,* Fusarium sambucinum* or *Phytophthora infestans* at both high and reduced water activities^1^, (3) evaluate biocontrol potential – whether negative or positive – via the quantitation of inhibition coefficients for potato tuber and *in vitro* assays and (4) investigate the mechanistic basis for any stimulation of potato pathogen vigour and proliferation.

## Results and discussion

Microbial strains with potential to control potato pathogens were isolated and characterized via a sequence of sampling/isolation steps, *in vitro* and *in vivo* experimental assays, and analyses of data as illustrated in Fig. S1.

### Search for novel biocontrol agents

It has been recurrently demonstrated that the phyllosphere is a natural reservoir of novel biocontrol agents of plant pathogens. Highly competitive species of *Pantoea*,* Pseudomonas* and *Pichia*, for example, have been sourced from this habitat (Cray *et al*., 2013a and references therein). One environment targeted by the sampling campaign of the current study was the leaves of the *S. tuberosum* (potato) plant. Although microbial endophytes inhabit a niche that is insulated from invasion by most microbial species, evidence suggests that the former can nevertheless exhibit high levels of competitive activity (Compant *et al*., 2010). We therefore sampled both leaf surfaces (phylloplane) and interiors (Table 1). The small group of microbes that have a high competitive ability and are able to dominate phylogenetically heterogeneous communities (known as ‘microbial weeds’; Cray *et al*., 2013a; Oren and Hallsworth, 2014) includes some of the most potent biocontrol agents (Cray *et al*., 2013a, 2015a). Key traits of microbial weed species can include a robust stress‐biology, especially xerotolerance, as well as nutritional versatility. That said, both nutritional generalists (e.g. *Pseudomonas putida*) and nutritional specialists (e.g. *Lactobacillus* species) occur within the microbial weeds (Cray *et al*., 2013a).

**Table 1:** Source of the microbial strains obtained by sampling potato fields and potato stores in Northern Ireland, and obtained by cultivation on diverse culture media.^a^

Culture medium	pH; water activity	Leaf surface			Leaf interior			Soil	
		(Cara)	(Maris Piper)	(Sárpo Mira)	(Cara)	(Maris Piper)	(Sárpo Mira)	Off‐season potato field	Surface of potato tubers in storage^b^
**High a_w_**									
Carrot agar (CA)	7.63; 0.997	JC12GB98, JC12GB99, JC12GB100, JC12GB101, JC12GB102, JC12GB103	JC12GB87, JC12GB88, JC12GB89, JC12GB90, JC12GB91	JC12GB92, JC12GB93, JC12GB94, JC12GB95, JC12GB96, JC12GB97	JC12GB104	JC12GB84, JC12GB85, JC12GB86	None	None^c^	None^c^
Reasoner's 2A (R2A agar)	7.46; 0.997	JC12GB112, JC12GB113, JC12GB114, JC12GB115, JC12GB116, JC12GB117, JC12GB118	JC12GB120, JC12GB121, JC12GB122, JC12GB123, JC12GB124, JC12GB125	JC12GB105, JC12GB106, JC12GB107, JC12GB108, JC12GB109, JC12GB110	JC12GB119	JC12GB126	JC12GB111	None^c^	None^c^
Nutrient agar (NA)	7.46; 0.996	JC12GB26, JC12GB27, JC12GB28, JC12GB29, JC12GB30, JC12GB31, JC12GB32	JC12GB33, JC12GB34, JC12GB35, JC12GB36, JC12GB37, JC12GB38, JC12GB39, JC12GB40	JC12GB46, JC12GB47, JC12GB48, JC12GB49, JC12GB50, JC12GB51, JC12GB52, JC12GB53	JC12GB44	JC12GB41, JC12GB42, JC12GB43	JC12GB45	JC11GB19^d^, JC11GB20^d^	JC11GB14, JC11GB15, JC11GB16, JC11GB17, JC11GB18, JC11GB25
NA+antibiotics	7.38; 0.996	JC12GB132, JC12GB133, JC12GB134, JC12GB135, JC12GB136	JC12GB144, JC12GB145	JC12GB127, JC12GB128, JC12GB129, JC12GB130, JC12GB131	None	JC12GB137, JC12GB138, JC12GB139, JC12GB140, JC12GB141, JC12GB142, JC12GB143	None	JC11GB1^e^, JC11GB3^e^, JC11GB21^d^, JC11GB23^e^, JC11GB24^f^	JC11GB22
Potato dextrose agar (PDA)	6.10; 0.994	JC12GB66, JC12GB67, JC12GB68, JC12GB69, JC12GB70, JC12GB71, JC12GB72, JC12GB73, JC12GB74	JC12GB54, JC12GB55, JC12GB56, JC12GB57, JC12GB58, JC12GB59, JC12GB60, JC12GB61, JC12GB62, JC12GB63	JC12GB76, JC12GB77, JC12GB78, JC12GB78, JC12GB79, JC12GB80, JC12GB81, JC12GB82, JC12GB83	None	JC12GB64, JC12GB65	JC12GB75	JC11GB2^e^, JC11GB4^e^, JC11GB27^g^, JC11GB28^g^	JC11GB5, JC11GB6, JC11GB7, JC11GB26
**Intermediate a_w_**									
R2A agar+1.104 M NaCl	7.05; 0.962	JC12GB146, JC12GB147, JC12GB148, JC12GB149, JC12GB150, JC12GB151	JC12GB153, JC12GB154, JC12GB155, JC12GB156	JC12GB164, JC12GB165, JC12GB166, JC12GB167, JC12GB168, JC12GB169	JC12GB152	None	JC12GB162, JC12GB163	None^c^	None^c^
NA+1.104 M NaCl	7.27; 0.961	JC12GB192, JC12GB193, JC12GB194, JC12GB195	JC12GB197, JC12GB198, JC12GB199, JC12GB200, JC12GB201	JC12GB157, JC12GB158, JC12GB159, JC12GB160, JC12GB161	JC12GB196	None	JC12GB189, JC12GB190, JC12GB191	None^c^	None^c^
R2A agar+1.583 M glycerol	7.63; 0.958	JC12GB170, JC12GB171, JC12GB172, JC12GB173, JC12GB174, JC12GB175	JC12GB180, JC12GB181, JC12GB182, JC12GB183, JC12GB184, JC12GB185, JC12GB186	JC12GB18, JC12GB19, JC12GB20, JC12GB21, JC12GB22, JC12GB23, JC12GB24, JC12GB25	JC12GB176, JC12GB177, JC12GB178, JC12GB179	JC12GB187, JC12GB188	None	None^c^	None^c^
NA+1.583 M glycerol	7.36; 0.957	JC12GB8, JC12GB9, JC12GB10, JC12GB11	JC12GB13, JC12GB14, JC12GB15, JC12GB16, JC12GB17	JC12GB1, JC12GB2, JC12GB3, JC12GB4, JC12GB5	JC12GB12	JC12GB7	JC12GB6	JC11GB8^f^, JC11GB9^d^, JC11GB10^g^, JC11GB11^g^, JC11GB12^e^, JC11GB13^e^	None^c^
**Low a_w_**									
NA+3.183 M NaCl	7.20; 0.878	None	None	None	None	None	None	None^c^	None^c^
NA+3.679 M glycerol	7.34; 0.914	JC12GB205, JC12GB206, JC12GB207, JC12GB208, JC12GB209	JC12GB210, JC12GB211, JC12GB212, JC12GB213	JC12GB202, JC12GB203, JC12GB204	None	None	JC12GB6	None^c^	None^c^

a The total number of strains obtained was: 243; those underlined were selected for further study (see Results and discussion).

b Samples were from a tubers of potato cultivar Dunbar Standard stored for several weeks in an open barn (under a roof but otherwise open to the atmosphere) in County Down, Northern Ireland (GPS: 54° 33′ 15.98″ N 5° 41′ 16.3″ W). Isolates were obtained by swabbing potato tubers and streaking onto media; see Experimental procedures.

c Microbial growth was prolific on these media, so individual strains could not be identified/obtained.

d Samples were from privately run vegetable allotments near Belfast, County Down, Northern Ireland (GPS: 54° 35′ 29.78″ N 5° 41′ 50.99″ W); see Experimental procedures.

e Samples were from privately run vegetable allotments near Belfast (GPS: 54° 34′ 27.46″ N 5° 41′ 47.75″ W); see Experimental procedures.

f Samples were from privately run vegetable allotments near Lisburn, County Antrim, Northern Ireland (GPS: 54° 32′ 21.51″ N 6° 65′ 40.96″ W); see Experimental procedures.

g Samples were from privately run vegetable allotments near Belfast (GPS: 54° 30′ 42.87″ N 5° 53′ 28.1″ W); see Experimental procedures.

It is serendipitous that biocontrol agents frequently exhibit xerotolerance because this can, critically, enable pathogen control over a maximal window of humidity or water activity in the field (Hallsworth and Magan, 1994a, 1995; Cray *et al*., 2013a). We obtained a total of 143 isolates on high water activity media (0.997–0.994 water activity), 87 isolates on moderate water activity media (0.962–0.957 water activity) and 13 isolates on low water activity media (0.914–0.878 water activity) (Table 1). The majority of the isolates were obtained from the leaf surface, regardless of potato cultivar (Cara, Maris Piper or Sárpo Mira) or type of nutrient medium (Table 1). Interestingly, on high water activity media, more isolates were obtained from the leaf interior (16) of potato cultivar Maris Piper than for either Cara (3) or Sárpo Mira (3), whereas most isolates obtained from the leaf interior on moderate water activity media originated from Cara and Sárpo Mira (13 in total) rather than Maris Piper (three in total; Table 1). By contrast, only one isolate was obtained from the leaf interior by plating samples on low water activity media (JC12GB6). In total, 17 isolates were obtained from fallow potato‐field soils and 11 from stored potato tubers (Table 1).

Culture media used for plating the samples included nutrient agar (NA) which is pH‐neutral and favours growth of many bacteria (and some eukaryotes), NA+antibiotics to inhibit bacteria and enable emergence of eukaryotic microbes (especially those with longer doubling times), potato dextrose agar (PDA) that is slightly acidic and can favour saprotrophs (especially eukaryotic species), Reasoner's 2A (R2A) agar that favours oligotrophic species, carrot agar (CA) to select for species capable of growth on this medium that is important for cultivation of (and interaction assays with) *P. infestans*, and NA or R2A agar supplemented with osmolytes (Table 1). The osmolytes used for this purposed were NaCl, which favours halotolerant species, and glycerol, permissive for other types of xerotolerant species (Cray *et al*., 2013a; Stevenson *et al*., 2015a) (Table 1). Isolates were obtained on each medium type except for the low water activity medium NA+NaCl (0.878 water activity) (Table 1). In addition, some microbes from soil samples grew on low water‐activity media, though no distinct isolates (Table 2) were obtained from these.

**Table 2:** Phenotypic characteristics of the microbial isolates used in interaction assays with plant pathogens.^a^

Microbe^b^	Colony morphology (shape, margin, elevation, texture, appearance, pigmentation, optical property)	Cell morphology (width [μm], length [μm])	Gram stain^c^	Oxidase test^d^
JC12GB6	Circular, entire, flat, moderate, smooth, shiny, none, translucent	Rod (1.0, 3.0)	+	−
JC12GB7	Circular, curled, flat, moderate, rough, shiny, yellow, opaque	Yeast (3.5, 5.0)	Not applicable	−
JC12GB12	Circular, entire, flat, moderate, rough, shiny, yellow, opaque	Coccus (0.5, 0.5)	+	−
JC12GB13	Circular, entire, flat, moderate, smooth, shiny, yellow, opaque	Coccus (0.5, 0.5)	+	−
JC12GB14	Circular, entire, flat, moderate, smooth, shiny, yellow, opaque	Coccus (0.5, 0.5)	+	+
JC12GB28	Circular, entire, flat, moderate, rough, shiny, none, translucent	Rod (1.0, 2.0)	−	−
JC12GB29	Circular, entire, flat, rough, moderate, shiny, yellow, translucent	Rod (1.0, 2.0)	−	−
JC12GB34	Circular, entire, flat, rough, moderate, shiny, yellow, translucent	Rod (1.0, 2.0)	−	−
JC12GB35	Circular, curled, flat, moderate, rough, shiny, yellow, opaque	Rod (1.0, 2.0)	−	−
JC12GB36	Circular, curled, flat, moderate, rough, shiny, yellow, translucent	Rod (0.5, 1.5)	−	−
JC12GB42	Irregular, undulate, flat, smooth, moderate, dull, none, opaque	Rod (0.5, 2.0)	+	+
JC12GB43	Irregular, undulate, flat, smooth, moderate, dull, none, opaque	Rod (0.5, 2.0)	+	+
JC12GB44	Irregular, undulate, flat, smooth, moderate, dull, none, opaque	Rod (0.5, 2.0)	+	+
JC12GB47	Circular, curled, flat, moderate, smooth, dull, none, opaque	Rod (0.5, 2.0)	−	−
JC12GB48	Circular, entire, flat, moderate, smooth, shiny, none, opaque	Rod (1.0, 2.5)	−	−
JC12GB50	Circular, curled, flat, moderate, rough, shiny, yellow, translucent	Rod (0.5, 1.5)	−	−
JC12GB51	Circular, undulate, flat, moderate, rough, shiny, white, translucent	Rod (0.5, 1.5)	−	−
JC12GB54	Circular, entire, flat, smooth, moderate, shiny, burnt orange, opaque	Coccus (0.5, 0.5)	−	+
JC12GB58	Circular, curled, flat, moderate, rough, shiny, none, translucent	Rod (1.0, 2.0)	+	−
JC12GB61	Circular, curled, flat, moderate, smooth, shiny, none, translucent	Coccus (1.0, 1.0)	+	−
JC12GB64	Circular, entire, flat, moderate, smooth, shiny, burnt orange, opaque	Diplococcus (1.0, 1.0)	+	−
JC12GB65	Circular, entire, flat, moderate, rough, shiny, burnt orange, translucent	Coccus (0.5, 0.5)	+	+
JC12GB70	Circular, entire, convex, smooth, punctiform, moderate, shiny, yellow, translucent	Coccus (0.5, 0.5)	−	−
JC12GB73	Circular, curled, flat, moderate, rough, dull, pink, opaque	Yeast (2.0, 5.0)	Not applicable	−
JC12GB75	Circular, undulate, flat, moderate, smooth, dull, none, translucent	Rod (0.5, 1.5)	−	−
JC12GB78	Circular, curled, flat, moderate, rough, dull, none, translucent	Rod (0.5, 2.0)	−	−
JC12GB80	Circular, entire, flat, moderate, rough, shiny, none, translucent	Rod (1.0, 2.0)	+	−
JC12GB189	Circular, curled, flat, moderate, smooth, shiny, yellow, opaque	Coccus (1.0, 1.0)	+	−
JC12GB190	Circular, entire, flat, moderate, smooth, shiny, none, translucent	Coccus (1.0, 1.0)	+	−
JC12GB191	Circular, entire, flat, moderate, smooth, shiny, none, translucent	Coccus (1.0, 1.0)	+	−
JC12GB196	Circular, entire, flat, moderate, smooth, shiny, yellow, opaque	Coccus (1.0, 1.0)	+	−
JC12GB197	Circular, entire, flat, moderate, smooth, shiny, yellow, opaque	Coccus (1.0, 1.0)	+	−

a According to Wimpenny (1988).

b Strain designation indicates the person who isolated the microbe (JC = Jonathan A. Cray), year of isolation (12 = 2012), country of isolation (GB = UK), and an isolate number as used by Williams and Hallsworth (2009).

c ‘+’ indicates Gram‐positive bacterium, ‘−’ indicates Gram‐negative bacterium; see Experimental procedures.

d A positive result indicates the detection of cytochrome oxidase enzymes; see Experimental procedures.

Of the 243 isolates obtained, the original, phylogenetically heterogeneous cultures (Table 1) and the subsequent interaction assays between pure cultures of individual isolates and plant pathogens (*F. coeruleum*,* F. sambucinum* and *P. infestans*; see Experimental procedures) were analysed to identify isolates with the greatest biocontrol potential. Using this approach, 32 isolates were selected for further study based on criteria such as vigorous growth kinetics, motility and/or ability to maintain zones‐of‐inhibition against a plant pathogen or other microbes (Table 2). These consisted of 17 rod‐shaped bacterial isolates, 12 cocci bacterial isolates, one diplococcus bacterial isolate and two yeast isolates (Table 2). Of the bacterial isolates, 17 were Gram‐positive, and six were oxidase‐test positive; based on these, and the morphological characteristics each isolate was unique and presumably, therefore, also phylogenetically distinct (Table 2). These isolates were used for a series of interaction assays to determine ability to inhibit or, indeed, promote plant‐pathogenic strains of *F. coeruleum*,* F. sambucinum* and *P. infestans*. For this purpose, established biocontrol agents were used as comparators; these were bacterial strains of *Bacillus subtilis*,* Pantoea*,* Pseudomonas fluorescens* and *Streptomyces*, and a strain of the yeast *Pichia anomala* (see Experimental procedures).

### Biocontrol agent:plant pathogen interactions at high water activity

**Figure 1:**
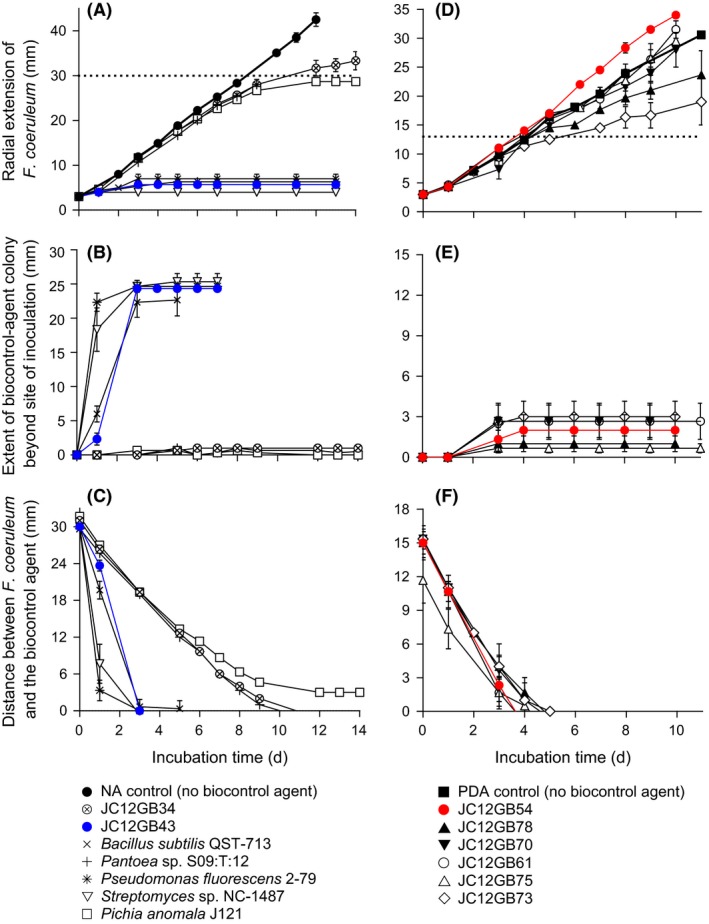
Interactions between *Fusarium coeruleum* and biocontrol agents on (A–C) NA and (D–F) PDA: (A and D) radial extension of *F. coeruleum* on the side adjacent to the biocontrol agent (dotted lines indicate the distance between *F. coeruleum* and biocontrol agent at the time of inoculation; (B and E) extent of colony of biocontrol agent beyond the initial zone‐of‐inoculation (over time) on the side adjacent to *F. coeruleum*; and (C and F) distance between *F. coeruleum* and potential biocontrol agent over time. Upon inoculation of NA and PDA,* F. coeruleum* and the biocontrol agents were placed 30‐and 13‐mm apart, respectively. Error bars indicate ± standard error.

**Figure 2:**
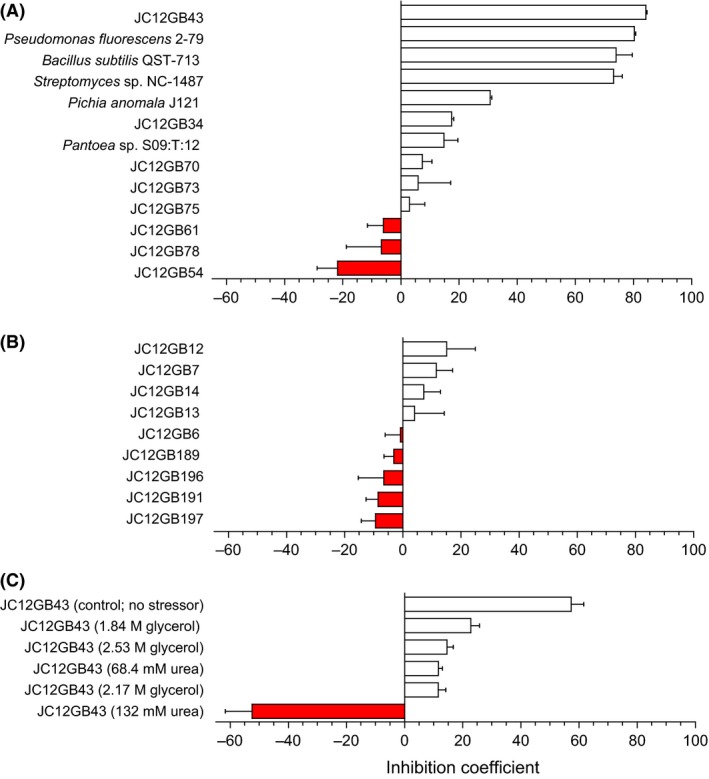
Inhibition coefficients for a range biocontrol agents used in inhibition assays against *Fusarium coeruleum* at (A) high water activity (NA or PDA) and (B) moderate water activity (NA+1.583 M glycerol or NA+1.104 M NaCl); (C) effects of glycerol or urea on the inhibition coefficient of JC12GB43 against *F. coeruleum*. Values for inhibition coefficients were determined as by Cray *et al*. (2015a,b) from the equation: Inhibition coefficient = [(100 − B) × 0.4] + [(100 − C) × 0.4] + [(100 − E) × 0.2] (see Table S2). Negative values (shown by red bars) indicate that colony development of the pathogen was promoted. Error bars indicate ± standard error.

**Figure 3:**
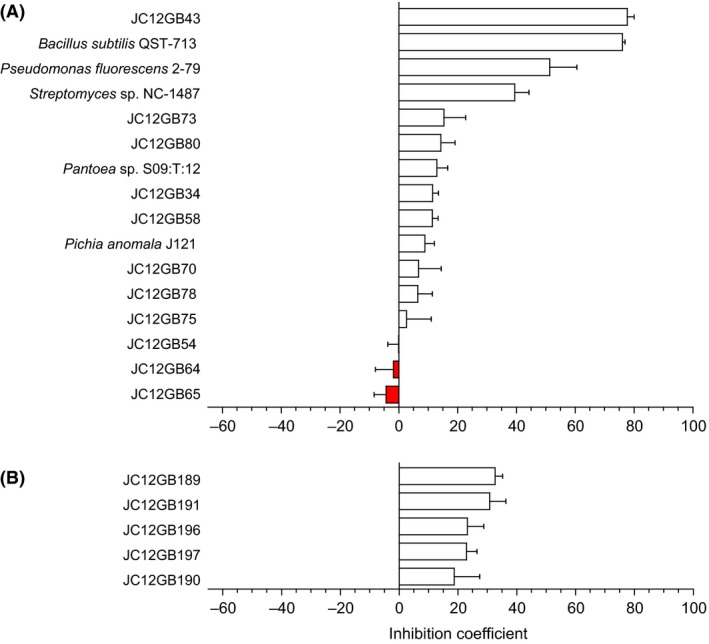
Inhibition coefficients for biocontrol agents used in inhibition assays against *F. sambucinum* at (A) high water activity (NA or PDA) and (B) moderate water activity (NA + 1.104 M NaCl). Values for inhibition coefficients were determined as by Cray *et al*. (2015a) from the equation: Inhibition coefficient = [(100 − B) × 0.4] + [(100 − C) × 0.4] + [(100 − E) × 0.2] (see Table S3). Negative values (shown by red bars) indicate that colony development of the pathogen was promoted. Error bars indicate ± standard error.

**Figure 4:**
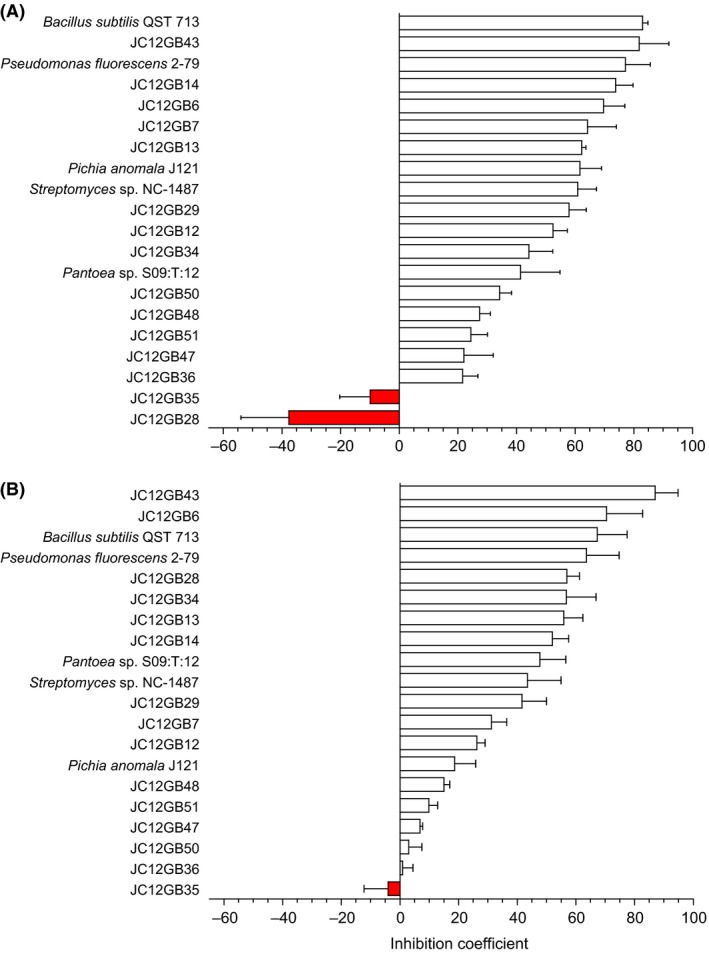
Inhibition coefficients for a range biocontrol agents used in inhibition assays against (A) *Phytophthora infestans* 10D2_5 on CA and (B) *P. infestans* 10LD3 on CA. Values for inhibition coefficients were determined as by Cray *et al*. (2015a,b) from the equation: Inhibition coefficient = [(100 − B) × 0.4] + [(100 − C) × 0.4] + [(100 − E) × 0.2] (see Table S5). Negative values (shown by red bars) indicate that colony development of the pathogen was promoted. Error bars indicate ± standard error.

**Figure 5:**
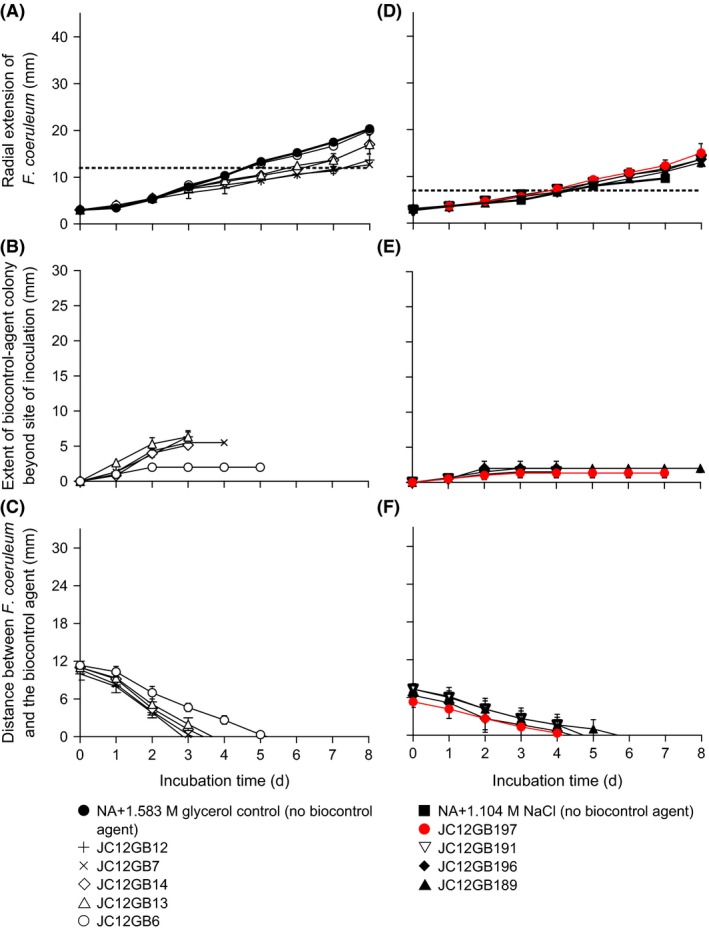
Interactions between *Fusarium coeruleum* and biocontrol agents on (A–C) NA+1.583 M glycerol and (D–F) NA+1.104 M NaCl: (A and D) radial extension of *F. coeruleum* on the side adjacent to the biocontrol agent (dotted lines indicate the distance between *F. coeruleum* and biocontrol agent at the time of inoculation); (B and E) extent of colony of biocontrol agent beyond the initial zone‐of‐inoculation (over time), on the side adjacent to *F. coeruleum*; and (C and F) distance between *F. coeruleum* and potential biocontrol agent over time. Upon inoculation on NA+1.583 M glycerol and NA+1.104 M NaCl, *F. coeruleum* and the biocontrol agents were placed 12‐ and 7‐mm apart, respectively. Error bars indicate ± standard error.

Interactions between biocontrol agents and plant pathogens take various forms. Some biocontrol agents, for example, are highly motile and/or can maintain a zone‐of‐inhibition in which the pathogen is unable to grow (Figs 1 and 5; Figs S2–S5; Tables S1–S4; Cray *et al*., 2015a). However, earlier studies demonstrated that effective pathogen‐inhibition cannot be predicted based solely on determinations of biocontrol agent motility and/or ability to maintain zones‐of‐inhibition (Cray *et al*., 2015a). Here, we used an inhibition coefficient (maximum potential value = 100) that was devised to quantify the potency of biocontrol agents against plant pathogens on solid surfaces, and is based on distance between biocontrol agent‐ and plant‐pathogen colonies, extent of the biocontrol agent lawn, and colony radius of the plant pathogen (Cray *et al*., 2015a). Colony area (as opposed to colony density) better reflects pathogen‐ and biocontrol‐agent distribution on a plant surface, so is pertinent to the risk of infection events (Cray *et al*., 2015a). Of the strains isolated in the current study, the most potent inhibitor on high water‐activity media was JC12GB43, regardless of the type of potato pathogen. This bacterial strain was able to minimize colony development of each plant‐pathogenic species, was motile and therefore spread considerably beyond the initial site of its inoculation, quickly minimized its distance from the plant pathogen, and managed to prevent further growth of the latter (Fig. 1; Figs S2–S4). Furthermore, *Bacillus* sp. JC12GB43 (isolated using NA at 0.996 water activity and yet xerotolerant; see later) was as potent as, or more potent than, the most effective commercial biocontrol agent, regardless of the plant pathogen. JC12GB43 was also significantly more inhibitory (*P* < 0.05) towards *F. coeruleum*,* F. sambucinum* and *P. infestans* 10LD3 than were the biocontrol agents *Pantoea* sp S09:T:12 and *P. anomala* J121 (Fig. 1; Figs S2 and S3): inhibition‐coefficient values for JC12GB43 were 84.2, 77.7, 87.0 and 83.0 when assessed against *F. coeruleum*,* F. sambucinum*,* P. infestans* 10LD3 and *P. infestans* 10D2_5 respectively (Figs 2A, 3A and 4; Tables S1–S4). Ribosomal Database Project and BLAST searches for partial 16S rDNA sequences revealed a definitive taxonomical affiliation for strain JC12GB43 as a member of the *Bacillus* genus (GenBank accession: KU359267; see Experimental procedures). The partial 16S rDNA sequence of strain JC12GB43 had ≥ 99% identity with, for example, *Bacillus methylotrophicus* strain MER_TA_47.1, *Bacillus* sp. 28592, *Bacillus siamensis* strain LYLB4, *Bacillus* sp. B4_UMNG, *Bacillus* sp. B3_UMNG and *Bacillus amyloliquefaciens* strain BCRh10. The identification of JC12GB43 as a *Bacillus* species is also consistent with characteristics in Table 2.

Of the strains isolated, others with potent biocontrol activity included JC12GB14, followed by JC12GB6, JC12GB7 and then JC12GB13, all of which were originally isolated on NA+glycerol (0.957 water activity; Figs 4 and 5; Fig. S4; Tables S1, S3 and S4). Overall, the inhibition‐coefficient values for isolates able to reduce growth of plant pathogens during the interaction assays ranged from 2.92 to 84.2, 2.64 to 77.7, 21.6 to 83.0 and 0.85 to 87.0 for *F. coeruleum*,* F. sambucinum, P. infestans* strain 10D2_5 and *P. infestans* strain 10LD3 respectively (Figs 2, 3, 4; Tables S1–S4). In addition, several of the potential biocontrol agents (JC12GB28, JC12GB35, JC12GB54), all of which were obtained from leaf surfaces (Table 1), actually *promoted* growth of the plant pathogens (and so exhibited negative values for inhibition coefficients) (Figs 1D–F, 2A, 3A and 4; Figs S2A–C and S4; Tables S1–S4). For instance, strain JC12GB54, which was not motile (see Fig. 1E) enhanced growth rate of *F. coeruleum* by 0.61 mm day^−1^ (Figs 1D and 2A). By contrast, strains JC12GB64, JC12GB65 and JC12GB75 were relatively ineffective as they were neither inhibitory nor able to promote growth of plant pathogens, regardless of species (Figs 1D–F, 2A, 3A; Fig. S2; Tables S1 and S2). Promotion of *F. coeruleum* was pronounced; growth rates were 202% that of the PDA+132 mM urea control (no biocontrol agent), when cultured on PDA+132 mM urea in the presence of *Bacillus* sp. JC12GB43 and 123% that of the PDA control (no biocontrol agent) on PDA in the presence of strain JC12GB54 (Fig. 2A and C; Tables S1 and S5). Growth rate of *P. infestans* 10D2_5 was 139% that of the CA control (no biocontrol agent) when cultured on CA and in the presence of strain JC12GB28 (Fig. 4A; Table S4). This acts as proof‐of‐principle that a potent biocontrol agents can promote proliferation of the pathogen.

**Figure 6:**
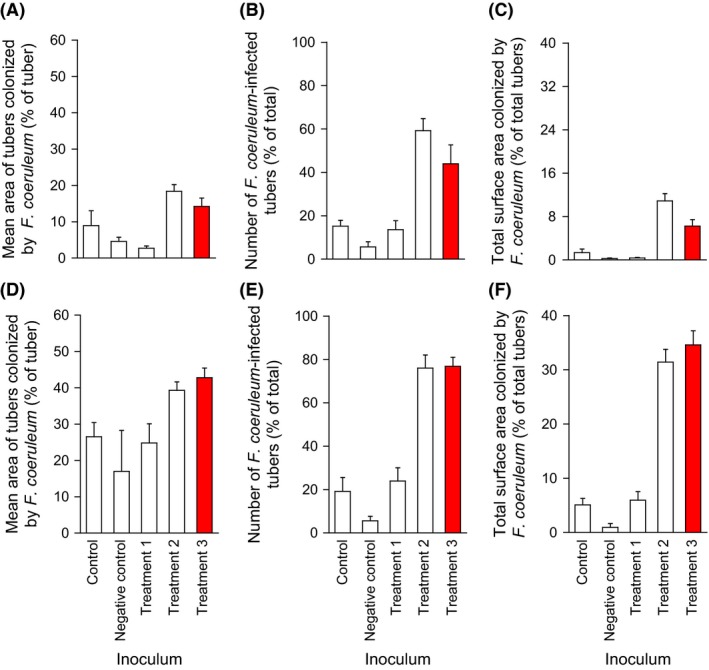
*Bacillus* sp. JC12GB43:*Fusarium coeruleum* interaction assays carried out on the wounded surface of Desirée (A–C) and Dundrod (D–F) potato tubers. Controls were wounded and inoculated using *F. coeruleum* only, and the negative control was not inoculated with either the pathogen or biocontrol agent. Treatments 1‐3 (T1‐3) were wounded and inoculated with *F. coeruleum* in the same way and then, after 24 h, also inoculated with *Bacillus* sp. JC12GB43 that had been cultured at high water activity (for Treatment 1, on NB at water activity 0.998) or reduced water activity (for Treatment 3, on NB+2 M glycerol at water activity 0.955). For Treatment 2 (T2), tubers were wounded and inoculated with *F. coeruleum* as described above and then, after 24 h, inoculated with NB+2 M glycerol only (i.e. no biocontrol agent). A suspension of *F. coeruleum* macroconidia (grown on PDA at 20°C for 17 days) was used for the potato‐pathogen inoculations (5 × 10^3^ ml^−1^) and a suspension of *Bacillus* sp. JC12GB43 cells was used for biocontrol‐agent inoculations (1.444 OD_600 nm_; taken from a late‐exponential phase culture). All tubers (250) were then stored for 100 days at ambient temperature, and without light, prior to assessment. For infected tubers, the mean surface‐area colonized by *F. coeruleum* (i.e. the area exhibiting dry rot) was quantified (A and D); the number of tubers colonized by *F. coeruleum* are expressed as a percentage of the total tubers (B and E); and the surface area of tubers colonized by *F. coeruleum* is also expressed as a total of all (infected plus non‐infected) tubers (C and F). Red bars indicate promotion of the pathogen which may be attributable to the biocontrol agent. Error bars indicate ± standard error.

Based on this finding, a parallel series of assays were carried out on potato tubers to characterize *Bacillus* sp. JC12GB43:*F. coeruleum* interactions in the context of their *in situ* ecology (see Experimental procedures). For the high water‐activity inoculum of *Bacillus* sp. JC12GB43 there was, however, neither any discernible stimulation of *F. coeruleum* nor prevention of tuber infection (*P *>* *0.05 for mean area of tubers infected, and mean number of tubers infected and total area of tubers infected) (Fig. 6). By contrast, the results of *in‐situ* assays carried out using low water‐activity inoculum did impact the degree of tuber infection by the pathogen (see below).

### Biocontrol agent:plant pathogen interactions at reduced water activity

For most biological phenomena and biotechnological processes, including biological control, sub‐optimal water availability can be a key limiting factor (Hallsworth and Magan, 1995; Teixidó *et al*., 2006; Cray *et al*., 2013a; Stevenson *et al*., 2015a,b). In order to carry out interaction assays to determine biocontrol potential at reduced water activity (which also simulates low‐humidity conditions in the field), we first assessed tolerance of low water activity for the plant pathogens. For this purpose, media were supplemented with a range of chemically diverse osmolytes including biologically permissive solutes, such as the compatible solutes proline, glycerol or sucrose (Williams and Hallsworth, 2009; Cray *et al*., 2013a), and what is generally considered the most biologically permissive salt, NaCl (Kar *et al*., 2015; Stevenson *et al*., 2015a) (Fig. S6). Although a small number of *Aspergillus flavus* strains (and closely related plant pathogens) are strongly xerotolerant (limit for hyphal growth ~0.775 water activity; Sanchis and Magan, 2004), the *Fusarium* strains under study were incapable of growth at 0.800 water activity, and *Phytophthora* strains were only able to grow at ≥ 0.960 water activity (Fig. S6). Although the *F. coeruleum* and *F. sambucinum* did not exhibit high levels of xerotolerance, they were able to grow down to ~0.847 and ~0.860 water activity, respectively, on most media and so could be used for interaction assays with biocontrol agents that had been isolated on moderate‐ and low water‐activity media (Table 1). With the sole exception of extreme, obligate halophiles (water‐activity limits in the range 0.650 to 0.610; Stevenson *et al*., 2015a), the most xerotolerant bacteria can only grow, and retain metabolic activity, down to 0.850 to 0.800 water activity, depending on species, whereas some fungi can do so in the range 0.690 to 0.605 (Williams and Hallsworth, 2009; Stevenson *et al*., 2015a,b). Indeed, the structures of some fungi hold various records for tolerance to extreme biophysical conditions (Cray *et al*., 2013a and references therein; Stevenson *et al*., 2015a,b; Wyatt *et al*., 2015a,b). It is, however, fortuitous that most plant pathogens are incapable of growth at ≤ 0.800 water activity (see below). It is nevertheless imperative to match the xerotolerance of a biocontrol agent to that of the pathogen because water availability is a key determinant of successful biocontrol whether *in vitro* or in the field (Hallsworth and Magan, 1994a, 1995; Magan, 2006; Rangel *et al*., 2015).

**Figure 7:**
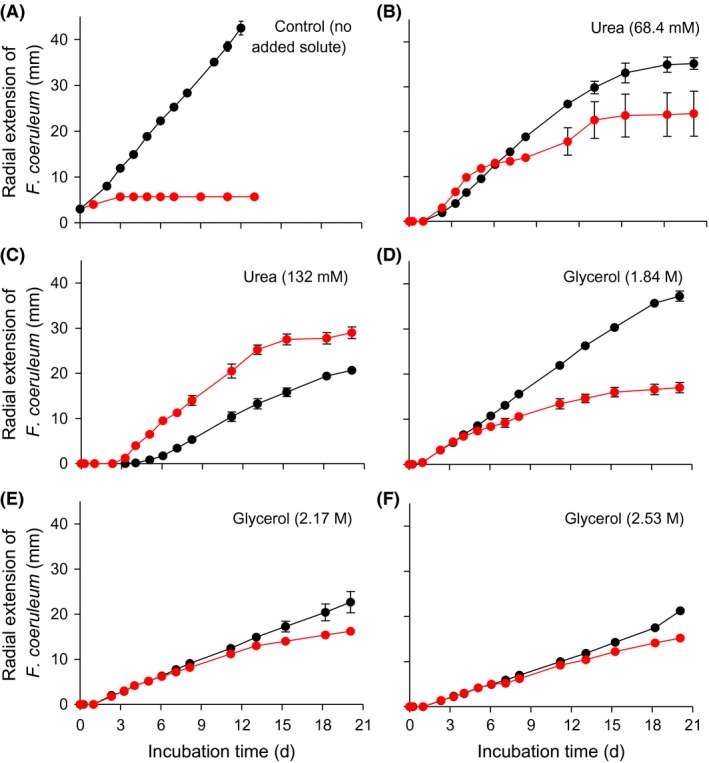
Radial extension of *Fusarium coeruleum* colonies when cultured alone (●) and during inhibition assays with *Bacillus* sp. JC12GB43, (●): on (A) PDA with no added solute; (B and C) PDA supplemented with urea; and (D–F) PDA supplemented with glycerol, at 20°C. Urea was incorporated into media at concentrations of 68.4 mM (b) and 132 mM (C); glycerol was incorporated at concentrations of 1.84 M (D), 2.17 M (E) and 2.53 (F). *Fusarium coeruleum* and *Bacillus* sp. JC12GB43 were inoculated 15‐mm apart, and error bars indicate ± standard error.

Interaction assays were carried out on media supplemented with solutes known to be permissive for growth of the biocontrol agents (Table 1); i.e. glycerol or NaCl. The media used for this purpose were NA+1.583 M glycerol (0.958 water activity) and NA+1.104 M NaCl (0.961 water activity); each of the biocontrol agents assayed (JC12GB6, JC12GB7, JC12GB12, JC12GB13, JC12GB14, JC12GB189 JC12GB190, JC12GB191, JC12GB196 and JC12GB197) had been isolated using one of these media (Fig. 5; Fig. S5). On these reduced water‐activity media, both the colony expansion of biocontrol agent strains, and radial growth rates of potato pathogens were decreased (Fig. 5) relative to those on high water‐activity media (Fig. 1). Importantly, despite the low water activity, the colonies of biocontrol agent strains JC12GB7, JC12GB12, JC12GB13 and JC12GB14 expanded rapidly (Fig. 5B), indicating potential for effective biocontrol at reduced water availability. Furthermore, these four isolates achieved medium coverage by forming a bacterial lawn (Fig. 5B). However, the biocontrol strains JC12GB12 and JC12GB7 (Fig. 5A) exhibited poor inhibition coefficients under these conditions; in the range 10–16 (Fig. 2B; Table S1). Interestingly, strains assayed on glycerol‐supplemented media were generally inhibitory to *F. coeruleum*, whereas those assayed on NaCl‐supplemented media induced a slight promotion of fungal growth (Fig. 5A and D). *Bacillus* sp. JC12GB43:*F. coeruleum* interaction assays using *Bacillus* inocula that had been cultured at 2 M glycerol (the chaotropic activity and water activity of the *Bacillus* pre‐culture medium = 3.28 kJ kg^−1^ and 0.955 respectively), i.e. Treatment 3 was carried out to determine the inhibitory potency of this biocontrol agent during the infection process on the potato tuber. Surprisingly, however, there was a promotion of the tuber *F. coeruleum* infection of tubers for treatments inoculated with the *Bacillus* sp. JC12GB43, 2‐M glycerol inoculum (Fig. 6). This promotion of the pathogen colony ranged from 65% to 570% relative to the level of infection observed in the control (control tubers were wounded and inoculated with *F. coeruleum*; no biocontrol agent) (Fig. 6). Although NB+glycerol inoculations were also of benefit to the pathogen (Fig. 6), this NB+2 M glycerol treatment (Treatment 2) had not been used as a culture medium and so did not have depleted levels of glycerol (some of which can be lost through evaporation during the incubation period, thereby increasing the water activity). The low water activity of Treatment 2, therefore, may have stressed and weakened the potato cells, slowed wound‐healing and thereby enhanced pathogen infection (wounded potato cells are highly metabolically active and susceptible to abiotic stresses; Morelli *et al*., 1998). Furthermore, the outcome for Treatment 2 in the *in‐vivo* assay was the converse of the outcome observed for the *in‐vitro* assay (Fig. 7D–F; Table S5). The *in‐vivo Bacillus* sp. JC12GB43:*F. coeruleum* interaction assay does, nevertheless, raise the possibility that formulation of biocontrol agents in either glycerol or other low molecular‐mass solutes (Torres *et al*., 2003; Bora *et al*., 2004; Patiño‐Vera *et al*., 2005) may potentially reduce or even reverse the biocontrol potential of microbial agents used in the field; more work is needed to confirm this.

*Fusarium coeruleum* is apparently osmotically stressed at water activities of ≤ 0.980 (see Fig. S6A), and so glycerol‐supplemented media/inoculum (known to boost growth rates of various fungi; Hallsworth and Magan, 1994b; Williams and Hallsworth, 2009) may have provided a ready source of compatible solute, in the form of glycerol, for use in osmotic adjustment (Figs 5A and 6). On NaCl‐supplemented media, we hypothesized that necromass from the relatively fast‐growing colonies of biocontrol agents provides a source of compatible solutes to cells of *F. coeruleum* that is not available to those of the NA+1.104 M NaCl control (no biocontrol agent) (Fig. 5D). This is consistent with earlier studies which demonstrated that bacterial metabolites can be taken up by fungi thereby promoting growth of the latter. For instance, Hildebrandt *et al*. (2006) reported enhanced growth of *Glomus intraradices* in the presence of the bacterium *Pseudomonas validus*, and provided evidence that raffinose produced by the bacterium acted as a stimulant for the fungus. Other studies also report that bacterial metabolites can enhance growth of fungal plant pathogens (Vespermann *et al*., 2007; Kai *et al*., 2008), though there is a paucity of information on whether these growth‐promoting activities can change to inhibitory activities under modified environmental conditions.

### Mechanisms by which biocontrol agents can promote fungal growth

Microbial habitats are typically characterized by multiple, dynamic stress parameters (Cray *et al*., 2013a; Harrison *et al*., 2013, 2015; Alves *et al*., 2015). Those located within the phyllosphere are prone to high‐temperature stress and dilution stress caused by hypo‐osmotic conditions (parameters that can destabilize macromolecular systems), depending on precipitation and other climatic factors. Potato‐leaf endophytes and phyllosphere microbes can also be exposed to a range of plant metabolites including complex aromatic substances (terpenoids, phenolics, alkaloids, etc.; Karamanoli, 2002) and, therefore, often exhibit catabolic pathways to utilize these as substrates (Nelson *et al*., 2002; Nikel *et al*., 2014). Such substances are typically potent chaotropic solutes, or hydrophobic stressors which also have chaotropicity‐mediated modes‐of‐inhibitory action; furthermore, volatile metabolites produced by microbial cells have similar modes‐of‐action (Hallsworth *et al*., 2003a; Bhaganna *et al*., 2010, 2016; Cray *et al*., 2013a,b, 2014, 2015a,b; Ball and Hallsworth, 2015). Microbes in the phyllosphere can therefore be exposed to high levels of chaotropicity which can also be compounded by applications of urea and other chaotropic plant fertilizers to the leaf surface (Krogmeier *et al*., 1989). Many phyllosphere species exhibit a considerable degree of chaotropicity tolerance (Hallsworth *et al*., 2003a; McCammick *et al*., 2010; Bhaganna *et al*., 2010; Cray *et al*., 2013a), a characteristic which is likely achieved, in part, through production of kosmotropic compatible solutes. Glycine, trimethylamine oxide, proline, betaine, ectoine, trehalose, mannitol and sorbitol (each of which can act as a compatible solute) are more polar than water, have a substantial hydration shell, and tend to be excluded from the aqueous phase of macromolecular systems, and their kosmotropic activities are −14.2, −25.9, −5.76, −25.5, −16.6, −10.6, −6.69 and −6.80 kJ kg^−1^ M^−1^ respectively (Cray *et al*., 2013a; Alves *et al*., 2015). *In‐vitro* studies have provided proof‐of‐principle that the chaotropic activities of urea, NH_4_NO_3_, phenol or MgCl_2_ can be mitigated, or even completely neutralized, by kosmotropic compatible solutes, including trimethylamine oxide, dimethylsulfoxide, proline, betaine, sorbitol and trehalose (Alves *et al*., 2015).

**Figure 8:**
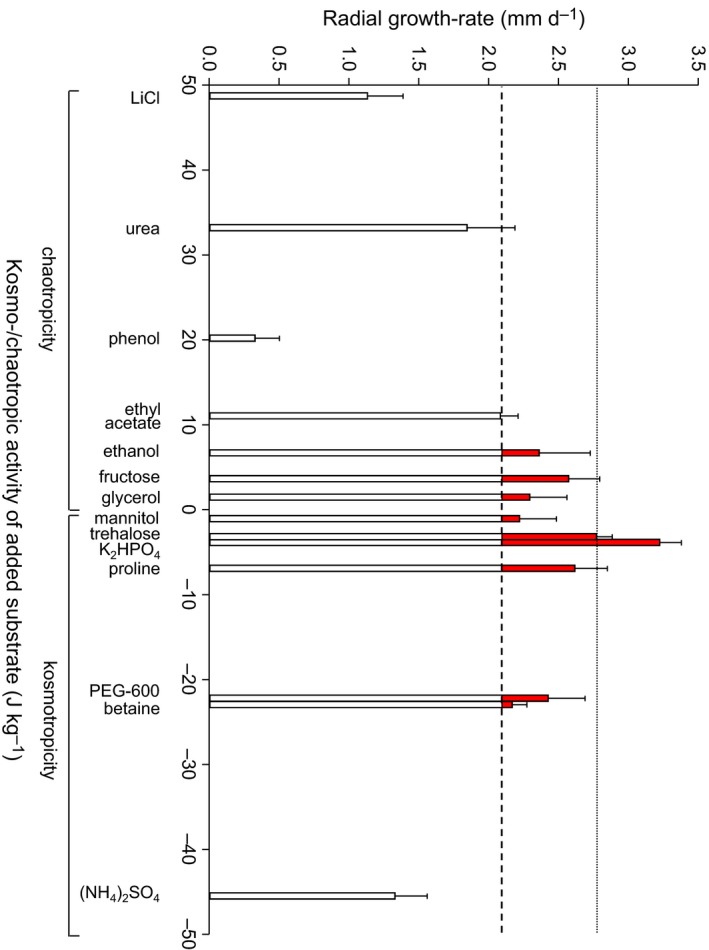
Radial growth rates of *Fusarium coeruleum* on PDA+132 mM urea supplemented by exogenous addition of solutes to modify medium kosmotropicity (positive values) and chaotropicity (negative values); see Table S6. Each solution contained either a compatible solute or other substance (Table S6) and was added (200 μl) to a 9 × 9 mm square well located 15 mm from the edge of the plug used for inoculation with the pathogen. The dotted line indicates radial growth rate of the control (PDA+132 mM urea, with sterile distilled water [200 μl] added to the well); the dashed line indicates radial growth rate of *F. coeruleum* when grown with *Bacillus* sp. JC12GB43 (placed 15‐mm apart) on PDA+132 mM urea; red bars indicate promotion of *F. coeruleum* growth rate relative to that of the control. Error bars indicate + standard error.

**Figure 9:**
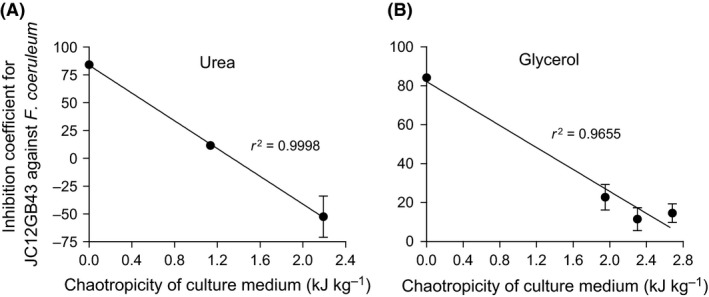
Effect of chaotropicity on the inhibition coefficient of *Bacillus* sp. JC12GB43 against *Fusarium coeruleum*. Interaction assays were conducted on PDA supplemented with the chaotropic solutes urea (A) or glycerol (B). Linear regression analyses were conducted using GraphPad Prism (Version 5).

**Figure 10:**
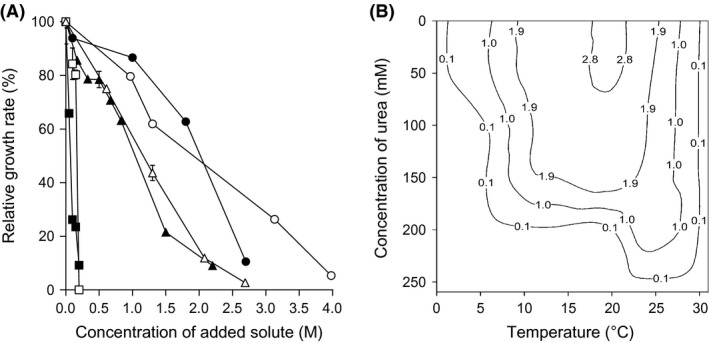
Growth phenotypes of *Fusarium coeruleum* and *Bacillus* sp. JC12GB43: (A) growth rates of *F. coeruleum* (open symbols) on PDA and *Bacillus* sp. JC12GB43 (closed symbols) in NB at their temperature optima (20°C and 30°C respectively) with urea (squares), glycerol (circles) or NaCl (triangles) over a range of concentrations, expressed as a percentage of the growth rate of the control (no added solutes); and (B) isopleth profile for radial growth rate of *F. coeruleum* over a range of temperatures and urea concentrations on PDA without (control; 0.996 a_w_) or with added urea. Isopleth contours indicate radial growth rates (mm day^−1^) determined by growth assays (see Fig. S7) and were plotted using Sigmaplot Version 8.0.

We hypothesized that compatible solutes can determine the outcome of biocontrol agent:plant pathogen interactions under stressful, macromolecule‐disordering conditions. Urea can stress microbial systems, reduce or prevent metabolism, and can be lethal like substances and parameters that increase entropy of macromolecular systems (e.g. see Fig. 8; Hallsworth *et al*., 2003a; Bhaganna *et al*., 2010; Cray *et al*., 2015b). As for previous studies, urea was used here as a model chaotrope (Hallsworth *et al*., 2003a, 2007; Bhaganna *et al*., 2010; Bell *et al*., 2013; Cray *et al*., 2013b; Alves *et al*., 2015). We sought to determine whether the outcome of interactions between the *Bacillus* (JC12GB43) and *F. coeruleum* vary according to chaotropicity. Interaction assays were carried out on PDA (control), PDA+68.4 mM urea, PDA+132 mM urea, PDA+1.84 M glycerol, PDA+2.17 M glycerol and PDA+2.53 M glycerol (glycerol becomes mildly chaotropic at molar concentrations). The growth rate of *F. coeruleum* alone (no added biocontrol agent) on PDA (no stressor added) was 3.80 mm day^−1^, and that for *F. coeruleum* in the presence of *Bacillus* sp. JC12GB43 was 1.33 mm day^−1^ (Fig. 2C; Table S5). On media with added urea, the growth rate of the plant pathogen in the presence of the biocontrol agent increased in proportion to the increase in chaotropicity. Indeed, on media supplemented with the highest urea concentration (132 mM), the biocontrol agent actually promoted growth rate of *F. coeruleum*, and did so by 102% relative to that of the PDA+132 mM urea control (no biocontrol agent). The negative inhibition coefficient value for the interaction assay between *Bacillus* sp. JC12GB43 and *F. coeruleum* on 132 mM urea media was −52.5; this inhibition‐coefficient value was significantly different (*P* < 0.05) to the inhibition coefficients for all other treatments (Figs 2C and 7C; Table S5) indicating a dramatic increase in pathogen growth rate. A similar trend was seen for glycerol‐supplemented media where the growth rates of controls (no biocontrol agent added) decreased with increasing glycerol concentrations, whereas those of the biocontrol agent remained relatively high (Fig. 7D–F). Inhibitory activity of *Bacillus* sp. JC12GB43 against *F. coeruleum* decreased as chaotropicity of the culture medium increased (Fig. 9). Urea (132 mM) alone does not promote growth of *F. coeruleum*, as shown in Fig. 10A. A recent study of MgCl_2_ (a chaotropic stressor like urea) has shown that viability of two *Mycobacterium* strains is only reduced at salt concentrations (1.5–3.5 M, depending on strain) that are beyond the window for growth; the upper MgCl_2_ concentration limit = 1 M for both strains (Santos *et al*., 2015). It seems unlikely, therefore, that 132 mM urea (which only caused 75% inhibition of *Bacillus* sp. JC12GB43 growth rate; Fig. 10A) adversely impacted viability of the biocontrol agent. Furthermore, studies of diverse bacterial species indicate tolerances towards urea close to 1 M (Cray *et al*., 2013a; Santos *et al*., 2015), whereas many enzyme systems are functionally stable at molar concentrations (Alves *et al*., 2015).

An isopleth growth profile was constructed for the parameters urea and temperature (see Experimental procedures) to determine whether *F. coeruleum* was chaotrope‐stressed in elevated urea concentrations at the incubation temperature (20°C) (Fig. 10; Fig. S7). This confirmed that optimum growth rate at 20°C is obtained between 0 and 50 mM urea, and that at 132 mM urea and 20°C, the growth rate of *F. coeruleum* reduced by 18% (Fig. 10A; Fig. S7), indicating that the pathogen was stressed. There is a sharp decline of urea tolerance at high temperatures (25–30°C) which contrasts with increased chaotrope tolerance at temperatures below 10°C (i.e. increased tolerance to low temperature in the presence of the chaotrope) (Fig. 10B; Fig. S7). This finding is consistent with reports of interactions between temperature and other chaotropic substances in relation to the growth windows of other microbial species (Hallsworth, 1998; Chin *et al*., 2010; Rummel *et al*., 2014; Cray *et al*., 2015b) and corroborates with the chaotropic mode‐of‐action as a stressor. For glycerol, a similar trend was observed (the growth rate of *F. coeruleum* alone declined more rapidly than that of *F. coeruleum* cultured in the presence of *Bacillus* sp. JC12GB43), although the effect was qualitatively less pronounced (Fig. 9; Fig. S7). Below 4 M, glycerol retains properties of a protectant and is a less potent chaotrope than urea, so this result was as expected (Brown, 1990; Williams and Hallsworth, 2009; Cray *et al*., 2013b).

Compatible solutes are primarily utilized by microbial cells for osmotic adjustment at low water activity, and/or to stabilize the macromolecular systems of pathogen cells under conditions that are otherwise disruptive (e.g. chaotropic substances, high temperature, dilution stress, hydrophobic stressors) (Elevi Bardavid *et al*., 2008; Chin *et al*., 2010; Cray *et al*., 2013a). At high solute concentrations, and upon osmotic up‐shock or down‐shock, bacterial cells release compatible solutes (Fischel and Oren, 1993; Schleyer *et al*., 1993; Sauer and Galinski, 1998; Halverson *et al*., 2000; Schubert *et al*., 2007; Hoffmann *et al*., 2012), so we hypothesize that *Bacillus* sp. JC12GB43 may release compatible solutes on urea‐supplemented media (Figs 7 and 8) and thereby enhance growth of the adjacent *F. coeruleum* colony. In order to test the plausibility of this hypothesis that fungal growth can be enhanced when under chaotropic conditions by compatible solutes of bacterial origin, we first quantified the growth rate of *F. coeruleum* under chaotropic conditions on PDA+132 mM urea (control: chaotropic activity of 2.191 kJ kg^−1^) as well as on the same medium with kosmotropic compatible solutes (betaine, proline, trehalose or mannitol), other kosmotropic substances (ammonium sulphate, potassium phosphate dibasic or PEG 600) or chaotropic substances (glycerol, fructose, ethanol, ethyl acetate, urea or phenol) added in a well located 15 mm from the inoculation plus (see Experimental procedures). At 132 mM urea, the growth rate of *F. coeruleum* is 45.0% less than that observed in the absence of added urea (Fig. 8; Table S5). Generally, neutral and mildly kosmotropic compatible solutes or osmolytes promoted growth rates of chaotropicity‐stressed *F. coeruleum*, and did so by up to 54.4% (Fig. 8; Table S6). Compatible solutes, therefore were able to completely mitigate the chaotropic effect of the added urea. Significantly, the addition of moderately chaotropic solutions (i.e. > 11 kJ kg^−1^) resulted in further inhibition of *F. coeruleum* growth rate (Fig. 8; Table S6). These data (in Fig. 8; Table S6) are consistent with the hypothesis that diffusion of bacterial metabolites through the nutrient medium can promote *Fusarium* growth rate. In addition, they provide proof‐of‐principle that kosmotropic substances can mitigate specific types of stress in plant pathogens. The finding is also consistent with studies of other biological processes where macromolecule‐stabilizing and macromolecule‐destabilizing factors mitigate stresses induced by each other, for both saline (Hallsworth *et al*., 2007; Rummel *et al*., 2014; Stevenson *et al*., 2015a; Yakimov *et al*., 2015) and non‐saline systems (Hallsworth *et al*., 2003b; Williams and Hallsworth, 2009; Bhaganna *et al*., 2010; Chin *et al*., 2010; Alves *et al*., 2015; Cray *et al*., 2015b).

### Implications for microbial ecology in agricultural systems and concluding remarks

Novel biocontrol agents were obtained by sampling potato‐associated microbial habitats, and the most potent of these proved to be *Bacillus* sp. JC12GB43. Tolerance to reduced water activity for strain JC12GB43 was considerably greater than that of *P. infestans*, and was close to that of *F. coeruleum* and *F. sambucinum*, suggesting that this biocontrol strain can compete with each of these plant pathogens regardless of environmental fluctuations in water availability and across all or most of the water‐activity windows for these plant pathogens. *Fusarium* species act as necrotrophs requiring wounds or other entry points into the tuber, whereas *P. infestans* is a hemibiotroph that requires living tissue or is able to penetrate intact tissue (although it enters tubers via eyes of lenticels). There is no evidence that these pathogens can multiply using vapour‐phase water alone (see Rummel *et al*., 2014; Stevenson *et al*., 2015b) and, in the context of pathogen:potato interactions, both *Fusarium* and *Phytophthora* (like many fungi) can be exposed to water‐activity values ranging from 1 down to values well below their water‐activity minima (e.g. on the dry surface of a leaf or tuber; Cray *et al*., 2013a). Ironically, *Bacillus* sp. JC12GB43 that was isolated on high water‐activity media (Table 1) was effective against *Fusarium* at low water‐activity (Fig. 2C) as well as high water‐activity (e.g. Figs 2A, 3A and 4), and strain JC12GB6 that came from low water‐activity medium (Table 1) was highly inhibitory to *P. infestans* at high water‐activity (Fig. 4). Although *Bacillus* sp. JC12GB43 was capable of near‐absolute inhibition of these pathogens, under some conditions there was a profound stimulation of pathogen growth. For *in‐vitro* interaction assays, this resulted in negative inhibition coefficients as great as −52.5 (Figs 2, 3, 4) and for interaction assays carried out on potato tubers, there was a promotion of tuber infection by almost 600% (Fig. 6). This finding may relate to release of kosmotropic metabolites (such as compatible solutes) by bacterial cells or necromass, though there is a possibility that it could relate to production of kosmotropic polysaccharides (e.g. extracellular polymeric substances) in combination with additional, yet‐to‐be‐identified mechanisms. Further work is needed to disentangle the respective contributions of *Bacillus* and the glycerol within the inoculum, to further optimize pathogen control *in vivo*.

Interaction assays carried out at high‐ and reduced water‐activities indicate that each biocontrol agent is most effective under (a) specific set(s) of conditions. This would be expected given the remarkable sensitivity of microbes to minute changes in this parameter (± 0.001 water activity; Stevenson *et al*., 2015b). It may be desirable, therefore, to inoculate crop plants using mixtures of ecophysiologically distinct biocontrol agents containing strains which differ in their levels of metabolism, and inhibitory capability, in relation to water availability. Furthermore, formulation of biocontrol agents for use as inoculum in the field can facilitate manipulation of the extracellular milieu once applied to the plant surface. Manipulation of compatible solutes can be used to enhance the growth windows of biocontrol agents (see above). For instance, hygroscopic substances can be added to enhance moisture retention, solutes could be used to reduce water activity, or compatible solutes in order to optimize xerotolerance. Such approaches, however, would have to be tailored for each biocontrol agent, plant pathogen, crop plant and/or set of environmental conditions. The need for bespoke, knowledge‐based biocontrol strategies is even more pressing given that inappropriate manipulations of the system could inadvertently promote growth of the plant pathogen (e.g. Figs 6, 7, 8).

Recent studies of interactions between *Aspergillus niger* and *Bacillus subtilis* demonstrate that each microbe reduces the metabolic processes which normally underlie their respective antimicrobial activities; in other words, contact between these species results in relatively mutualistic behaviour (Benoit *et al*., 2014). These findings are consistent with the observed promotion of plant pathogens in the current study, and offer a tantalizing suggestion that mechanisms additional to the biophysical processes described above may also be at play during interactions between strains such as *Bacillus* sp. JC12GB43 and pathogens. This raises the possibility that such *Bacillus* strains could be used to boost fungal activity when needed, in the context of agriculture (e.g. fungal biocontrol agents or soil inocula) or industrial fermentations (e.g. Cray *et al*., 2015b).

We still know relatively little about the biophysics and cellular biology underlying interactions between microbes in their natural environments; yet it is the entire system (i.e. biocontrol strain–plant pathogen–plant–abiotic environment‐extant microbial community) that must be understood and then manipulated in order to achieve optimal biological control (Cray *et al*., 2013a). In addition, successful pathogen control requires microbes capable of withstanding fluctuations in water activity, pH, chaotropicity, temperature, UV, etc. that are also capable of producing potent antimicrobials (see Cray *et al*., 2013a; Rangel *et al*., 2015; Suryawanshi *et al*., 2015). The UV‐ and temperature‐tolerances of *Bacillus* sp. JC12GB43 require further study to determine its suitability as a commercial biocontrol agent. However, its capacity for role reversal, which can lead to enhanced vigour and increased proliferation of the pathogen, may not be unique to this strain. Indeed, it may be that most, even all, biocontrol agents can promote pathogen growth under some circumstances. The phenotypic plasticity of biocontrol agents can be exploited to enable successful biological control (Hallsworth and Magan, 1995; Cray *et al*., 2013a; Rangel *et al*., 2015). It is ironic, therefore, that phenotypic plasticity of biocontrol strains can potentially increase crop losses by stimulating the activities of plant pathogenic species. Furthermore, the findings of the current study indicate that biocontrol activity ought to be regarded as a mode‐of‐behaviour (that is dependent on prevailing conditions), and is not an inherent property *per se* of the biocontrol strain.

## Experimental procedures

### Sampling, isolation and characterization of ecophysiologically distinct potato‐associated microbes

Leaves of three main‐crop potato (*Solanum tuberosum*) cultivars were taken from plants growing in a field in County Antrim, Northern Ireland (54° 33′ 26.55″ N, 5° 56′ 48.10″ W). These cultivars were Cara, Maris Piper and Sárpo Mira; known to differ in their levels of partial resistance to foliar late blight (Lees *et al*., 2012). Sampling was carried out at 11:30 am on 14 August 2012; at the time of sampling there was no precipitation, 74% humidity and an ambient temperature of 18°C. Leaflets (~15 cm in length) were cut, placed into sterile 2.5 L polypropylene boxes and stored at 5°C until further processing. Microbes from the leaf surface were prepared by placing leaf tissue (3 g) into 250 ml Erlenmeyer flasks with 50 ml of sterile saline solution (0.8% w/v NaCl), and then agitating in a rotary shaker at 150 rpm at 22°C for 1 h. Microbes were released from the interior of the leaves following Kuklinsky‐Sobral *et al*. (2004). Briefly, leaves were washed in saline solution (see above), washed in 70% (v/v) ethanol for 1 min, sodium hypochlorite solution (2% w/w available chlorine) for 3 min, and 70% (v/v) ethanol for a further 0.5 min; and then two rinses were carried out using sterile distilled water. Aliquots (100 μl) of the final rinses were pipetted onto NA (Oxoid Ltd, UK) plates, which were then incubated and monitored for quality‐control purposes (to assess/confirm surface sterility). Surface‐sterilized plant tissue was then macerated using a sterile pestle and mortar, releasing aqueous endophytic material.

Leaf‐surface washes and leaf extracts were serially diluted and inoculated on to a range of media: NA (0.996 a_w_; a high‐nutrient medium that favours growth of bacteria); NA+0.1 g l^−1^ oxytetracycline hydrochloride and 0.1 g l^−1^ streptomycin sulphate (0.996 a_w_; a high‐nutrient, antibacterial medium); NA+1.104 M NaCl (0.961 a_w_), NA+1.583 M glycerol (0.957 a_w_), NA+3.183 M NaCl (0.878 a_w_) and NA+3.679 M glycerol (0.914 a_w_) (high‐nutrient, low water‐activity media); R2A agar (0.997 a_w_; Fluka Analytical, Switzerland; a low‐nutrient medium); R2A agar+1.104 M NaCl 0.962 a_w_, R2A agar+1.583 M glycerol (0.958 a_w_; low‐nutrient, low water‐activity media); PDA (0.994 a_w_; Fluka Analytical, Switzerland; low‐pH, high‐nutrient complex‐substrate medium); and CA (0.997 a_w_): 200 g l^−1^ freshly frozen baby carrots (CH5 2NW; Iceland Foods Ltd., UK) blended and filtered through muslin, 15 g l^−1^ agar (Sigma‐Aldrich, Steinheim, Germany; undefined medium) (see Table 1). A 100 μl aliquot of each leaf‐surface and leaf‐extract dilution was inoculated onto each medium and spread with a glass spreader. All plates were sealed in Parafilm^®^ and stored at 20°C for several days. Once sufficient microbial growth on the plates had occurred, individual and morphologically distinct colonies were scraped from the culture‐medium surface and suspended in 1 ml of 20% (v/v) glycerol solution in a 2 ml Eppendorf tube, then frozen at −80°C (Table 1).

Soil samples were taken from privately run, off‐season potato allotments in County Down and County Antrim, Northern Ireland between 9:15 am and 1:30 pm, 10 November 2011 (54° 34′ 27.46″ N, 5° 41′ 47.75″ W; 54° 30′ 42.87″ N, 5° 53′ 28.1″ W; 54° 35′ 29.78″ N, 5° 41′ 50.99″ W) and between 9:15 and 9:45 am on 11 November 2011 (54° 32′ 21.51″ N, 6° 65′ 40.96″ W); the prevailing weather conditions were 12°C, 89% relative humidity, cloud cover but no rain, wind 27 km h^−1^. In addition, swabs from tubers of potato cultivar Dunbar Standard Mollies, stored for several weeks in an open barn (under a roof but otherwise open to the atmosphere), were also taken between 10:45 and 11:00 am on 10 November 2011 (54° 33′ 15.98″ N, 5° 41′ 16.3″ W). Samples were collected from the top 10 cm of soil using a sterilized spatula and placed into Sterilin^™^ polypropylene 30 ml universal containers; sterile cotton wool swabs were to swab potato tubers, the former were then placed into a sterile 30 ml Universal. Soil samples (1 g) were mixed with sterilized saline solution (0.8% w/v NaCl), diluted (to make a series of 10‐fold dilutions), and the 10^−2^ and 10^−6^ dilutions (100 μl of each) used to inoculated a range of media (see below; Table 2); swabs were streaked directly onto a range of solid media (see below; Table 2).

Microbes were cultured on their respective isolation medium for 24 h at 20°C prior to phenotypic characterization. Colony shape, margin, elevation, texture, appearance, pigmentation and optical property was noted; Gram staining was conducted using a Gram Stain Kit (Safranin Counterstain; Atom Scientific, Manchester, UK), and presence of cytochrome oxidase enzymes was detected using TestOxidase^™^ (Pro‐Lab, Richmond Hill, ON, Canada; Table 2). The most inhibitory microbe, strain JC12GB43 (see below) was identified via amplification of partial 16S rDNA genes according to Weisburg *et al*. (1991). DNA was extracted from a culture grown in nutrient broth (NB) by pipetting 500 μl into 500 μl Eppendorf tubes and centrifuging (6600 *g*). The pellet was re‐suspended in sterile, nuclease‐free water (Fisher Scientific Ltd, Loughborough, UK), up to 500 μl and placed in a heating block (99°C, 15 min) to lyse cells, centrifuged (6600 *g*, 15 min), and the supernatant used as template DNA. The reaction mix of each PCR contained: 10× DreamTaq^™^ buffer 5, dNTP Mix, 2 mM each (4 μl), 27f primer (5′‐AGAGTTTGATCMTGGCTCAG‐3′), 25 μM (1 μl), 1492r primer (5′‐TACGGYTACCTTGTTACGACTT‐3′), 25 μM (1 μl), DreamTaq^™^ DNA polymerase, 5 U μl^−1^ (0.25 μl), DNA template (2.5 μl), sterile, nuclease‐free water (36.25 μl; final volume 50 μl) and reactions were carried out in 500 μl PCR tubes using a thermal cycle as described previously (Weisburg *et al*., 1991). Each PCR was checked for successful amplification by agarose gel electrophoresis using a 2% (w/v) agarose gel: 20 μl (15 μl of sample and 5 μl of Gel Loading Buffer; Sigma‐Aldrich) were added to each well and gels were run for 60 min at 120 V, 500 mA. Bands were excised using a sterile, disposable scalpel and the DNA extracted using a Zymoclean^™^Gel DNA Recovery Kit and concentration quantified using a NanoDrop^™^ 1000 UV/Vis spectrophotometer. Single‐stranded DNA samples were sequenced by DNA Sequencing & Services (MRC I PPU; College of Life Sciences, University of Dundee, Scotland, UK, www.dnaseq.co.uk) using Applied Biosystems Big‐Dye Ver 3.1 chemistry on an Applied Biosystems model 3730 automated capillary DNA sequencer and taxonomical relatedness was assessed using Ribosomal Database Project (Wang *et al*., 2007) and BLAST nucleotide searches. The sequence of strain JC12GB43 was deposited at the NCBI GenBank.

### Strains of commercially available biocontrol agents and potato pathogens

Cultures of potato pathogens were obtained from the Agri‐Food and Biosciences Institute (AFBI; Newforge Lane, Belfast, UK). These were: the dry‐rot potato pathogens *Fusarium coeruleum* BL1/10 T5, originally isolated from a tuber of the cultivar up‐to‐date in Belfast in January 2011; *Fusarium sambucinum*, which was originally isolated in Scotland and subsequently passaged through potato tubers in Belfast to confirm that it had retained its pathogenicity; and isolates of the late‐blight potato pathogens *Phytophthora infestans* 10LD3 (genotype 8_A1), which was isolated from *S. tuberosum* cultivar Maris Piper foliage in Maghera, County Londonderry, Northern Ireland in August 2010, and *P. infestans* 10D2_5 (genotype 13_A2) which was isolated from an R5 differential plant of *S. tuberosum* in Belfast in August 2010. Both of the *P. infestans* isolates were previously genotyped by Restriction Fragment Length Polymorphism analysis using the moderately repetitive probe RG57 (Goodwin *et al*., 1992).

Several microbes known to have inhibitory activity towards potato pathogens were also obtained and assayed for comparative purposes; to benchmark the biocontrol potential of novel strains. These were: *Bacillus subtilis* QST 713, the active biocontrol agent in the product Serenade^®^, a commercial product marketed for control of grey mould – *Botrytis cinerea* – of fruit and vegetables; *Pseudomonas fluorescens* 2‐79 (NRRL B‐15132) and *Pantoea* sp. S09:T:12 (NRRL B‐21104), biocontrol agents of *Fusarium* dry rot (Schisler *et al*., 1995; Slininger *et al*., 2007); *Streptomyces* sp. NC‐1487, a recombinant strain derived via genome shuffling, which is effective against *P. infestans* (Clermont *et al*., 2011); and *Pichia anomala* (J121), a yeast which originally isolated from moist wheat grain with antibacterial and antifungal activity (Schnürer and Jonsson, 2011).

### Inoculation of biocontrol agent:potato pathogen interaction assays and assessment of colony development parameters

Interaction assays for each biocontrol agent:potato pathogen combination were carried out by simultaneously inoculating media with biocontrol agents and plant pathogen, using 9‐cm Petri plates as described by Cray *et al*. (2015a). Media were also inoculated with the plant pathogen only (i.e. controls). The distances between the biocontrol agent and plant pathogen were adjusted according to the growth rate of the fungus on the medium (see Fig. S8) and for *F. coeruleum* were: 30 mm on NA and 13 mm on PDA (Fig. S8); 12 mm on NA+1.583 M glycerol and 7 mm on NA+1.104 M NaCl; and 15 mm on PDA+68.4 mM urea, PDA+132 mM urea, PDA+1.84 M glycerol, PDA+2.17 M glycerol, and PDA+2.53 M glycerol. For *F. sambucinum*, these inoculation distances were: 40 mm on NA and PDA; and 13 mm on NA+1.104 M NaCl. For *P. infestans* 10LD3 on CA inoculation distance was 25 mm; and for *P. infestans* 10D2_5 on CA was 16 mm. These inoculations were carried out by taking 5‐mm diameter agar plugs from the periphery of exponential‐phase plant‐pathogen cultures (that had been incubated at 20°C) and placing on the surface of the assay medium. Each biocontrol agent was inoculated onto culture media by placing a thin smear of cells, taken from overnight cultures using a wire loop (each had been grown at 20°C, on the same medium as the assay was carried out). Plates were sealed using Parafilm^®^, incubated at 20°C and assessed regularly over the 21‐day assessment period, or until the plant pathogen had reached the edge of the plate. The radius of each plant‐pathogen colony on the side adjacent to the biocontrol agent, the radius of the biocontrol agent colony on the side adjacent to the plant pathogen, and the distance between plant pathogen‐ and biocontrol agent colonies were measured using a graduated ruler as described previously (Hallsworth *et al*., 1998; Kashangura *et al*., 2006; Cray *et al*., 2015a). All assays were conducted in triplicate and mean values were used to construct the displays (Fig. 1).

### Determination of growth rates and calculation of inhibition coefficients

Radial extension of the plant pathogen on the side adjacent to the biocontrol agent was plotted versus time and used to calculate radial growth rates; i.e. growth rate A (Tables S1–S5). Growth‐rate values were also determined as a percentage of the radial extension rate of each plant pathogen in control cultures; i.e. value B (Figs S2–S5; Tables S1–S5). The radial measurements of the plant‐pathogen colony on the side adjacent to the biocontrol agent were also used to calculate the distance travelled by the plant pathogen as a percentage of the distance between the sites of inoculation; i.e. value C (Figs S2–S5; Tables S1–S5) as described by Cray *et al*. (2015a). For any plant pathogen which came into contact with the biocontrol agent, measurements of radial extension made after this time were used to determine plant‐pathogen growth rates in the zone‐of‐mixed culture; i.e. growth‐rate D (Figs S2–S5; Tables S1–S5). This value was also expressed as a percentage of the rate of radial extension of the plant pathogen in the control culture; i.e. value E (Figs S2–S5; Tables S1–S5). The inhibitory activity was then quantified according to the equation devised by Cray *et al*. (2015a):

$$\begin{array}{cc} \text{Inhibition coefficient} & =[(100-B)\times 0.4]\\ & \;+[(100-C)\times 0.4]\\ & \;+[(100-E)\times 0.2] \end{array}$$

where [(100 − B) × 0.4] represents a potential 40% contribution of distal inhibition (prior to contact) of growth rate of the plant pathogen; [(100 − C) × 0.4] represents a potential 40% contribution of prevention of development of the plant pathogen colony in the vicinity of the biocontrol agent; and [(100 − E) × 0.2] represents a potential 20% contribution of ability to inhibit plant pathogen growth rate when in a zone‐of‐mixed culture. All assays were conducted in triplicate and data were analysed by one‐way ANOVA and Tukey's *post hoc* analyses using GraphPad Prism 5.01 (Figs 2, 3, 4; Tables S1–S5; see also Cray *et al*., 2015a).

### Determination of water‐activity limits and tolerance to chaotropic solutes

For determinations of water‐activity limits for growth of plant pathogens, preferred culture medium for each pathogen was first selected by culturing each on a range of nutrient media. *Fusarium coeruleum*,* F. sambucinum* and *P. infestans* 10LD3 and 10D2_5 were each grown on PDA, NA, NA+1.104 M NaCl, NA+3.183 M NaCl, NA+1.583 M glycerol, NA+3.679 M glycerol, R2A agar, R2A agar+1.104 M NaCl, R2A agar+1.583 M glycerol and Czapek Dox Agar; in addition *P. infestans* strains were also cultured on CA and Pea Agar (PA; 160 g l^−1^ blended Iceland Freshly Frozen Peas (CH5 2NW; Iceland Foods Ltd.), 15 g l^−1^ agar (Sigma‐Aldrich), 0.5 g l^−1^ sucrose). Agar plugs (5 mm diameter) were then taken from the periphery of exponential‐phase cultures, and used to inoculate each medium (one plug was placed centrally on each Petri plate). Plates were sealed using Parafilm^®^, incubated at 20°C and assessed throughout a 28‐day period or until the plant pathogen had reached the edge of the plate (visual inspection of plates after this period indicated that there was development of new colonies). The diameter of each colony was measured in perpendicular directions, and these values were used to calculate the mean radius (data not shown). Radial growth‐rate values were then plotted against time, and linear regressions were applied to the section of the curves corresponding to exponential growth (Fig. S8; as in Williams and Hallsworth, 2009; Marín *et al*., 2010; Cray *et al*., 2015a).

To determine the water‐activity limits, preferred media were selected: NA for *Fusarium* spp. and CA for *P. infestans* (note that, although *P. infestans* 10D2_5 has a higher growth rate on PA than CA [see Fig. S8], the opacity of PA interfered with growth rate determinations). The selected nutrient media (i.e. the controls) were also used to create a range of low water‐activity media by supplementing with NaCl, proline, glycerol or sucrose, over a range of concentrations (Fig. S6). Plates were inoculated, sealed, incubated and measured as stated previously. Radial growth‐rate values were plotted against time, and linear regressions were then applied to the section of the curves corresponding to exponential growth (Fig. S6). All stress‐tolerance assays were conducted in triplicate.

*Bacillus* sp. JC12GB43 cultured in NB (i.e. the NB control) and NB supplemented with a range of concentrations for each of the stressors glycerol, NaCl and urea (50 ml medium in a 250 ml Erlenmeyer flask, incubated at 30°C). Overnight cultures, adjusted to an OD_600_ of 0.5 by adding NB, were used to inoculate each flask (100 μl) which were then sealed with non‐absorbent cotton wool and covered in aluminium foil. Spectrophotometric readings were typically taken every hour, though less frequently for highly stressed cultures. All OD_600_ determinations were made in triplicate, and mean exponential growth rates (*k*) were calculated using GraphPad Prism 5.01.

The water activity of all media was determined empirically at 20°C using a Novasina Humidat IC II water activity machine fitted with an alcohol‐resistant humidity sensor and eVALC alcohol filter (Novasina, Pfäffikon, Switzerland) and employing standard precautions to ensure accuracy and reproducibility, as described previously (see Hallsworth and Nomura, 1999; Stevenson *et al*., 2015a). The instrument was calibrated in between measurements using saturated salt solutions of known water activity (Winston and Bates, 1960). Media water‐activity values were determined in triplicate; the variation of replicate values was within ± 0.001 (Stevenson *et al*., 2015a,b).

For *F. coeruleum*, urea tolerance was determined as described for assessment of water‐activity limits, but using urea as the stressor. Relative growth rates of *Bacillus* sp. JC12GB43 and *F. coeruleum* were determined by regarding the *k*‐values for growth on media with no added solute (i.e. their respective control cultures) as 100%, and calculating other growth rates relative to this.

### Construction of an isopleth growth profile for urea versus temperature tolerance of *F. coeruleum*

To determine urea:temperature interactions in relation to growth of *F. coeruleum*, inoculations were made onto PDA (i.e. the PDA control) and PDA+urea (from 50 to 300 mM), and these were incubated at temperatures of 0.3, 5.2, 11.6, 13.4, 19.8, 23.2 and 30.2°C. Plates were inoculated with a 5‐mm diameter agar plug taken from the periphery of an exponential‐phase *F. coeruleum* colony grown on PDA, and were sealed with Parafilm^®^. Colony measurements were made daily, over a 28‐day period (or until the colony reached the edge of the plate) and radial growth rates determined as described above (Fig. S7). These data were used to generate isopleth contours representing the *F. coeruleum* growth phenotype in relation to urea concentration versus temperature, as carried out previously for other microbes in relation to glycerol and temperature (Williams and Hallsworth, 2009; Stevenson and Hallsworth, 2014). All assays were carried out in triplicate.

### Assays for potential roles of compatible solutes as determinants of biocontrol agent: plant pathogen interactions

The possibility that biocontrol agents can promote growth of plant pathogens on chaotrope‐supplemented media (chaotropes are substances which destabilize macromolecular structures; Hallsworth, 1998; Cray *et al*., 2013b, 2015b; Ball and Hallsworth, 2015) by producing of macromolecule‐stabilizing (kosmotropic) compatible solutes was tested via two assays. Assay 1 was an interaction assay between *Bacillus* sp. JC12GB43 and *F. coeruleum* (see above) on: NA (i.e. the NA control), NA+68.4 mM urea, NA+132 mM urea, NA+1.84 M glycerol, NA+2.17 M glycerol and NA+2.53 M glycerol (glycerol becomes mildly chaotropic at molar concentrations; Cray *et al*., 2013b). The distance between biocontrol agent and plant pathogen at inoculation was 15 mm. Inhibition coefficients were calculated as described above and plotted in Fig. 2C, and plotted against chaotropicity of the medium in Fig. 10 (the latter was calculated based on values in Cray *et al*., 2013b); linear regression analyses were then conducted to test the hypothesis that chaotropic environmental conditions favour biocontrol activity and/or disadvantage the plant pathogen.

Assay 2 was carried out to determine whether the inhibition of *F. coeruleum* on NA+132 mM urea^2^ could by mitigated by kosmotropic compatible solutes or other kosmotropic substances. To this end, media were inoculated with *F. coeruleum* as for Assay 1 but (instead of inoculation with a biocontrol agent) a 9 × 9 mm square well was simultaneously made in the medium at 15 mm from the inoculation plug/colony. A solution (200 μl) was added to the well by pipette containing either a kosmotropic salt (ammonium sulphate, or potassium phosphate dibasic), relatively chao/kosmotropicity‐neutral compound (glycerol, proline or mannitol), and chaotropic compounds (lithium chloride, ethanol, urea, ethyl acetate, phenol or fructose). Solution concentrations were close to the limit of solubility for each substance (see Table S6). Plates were sealed with Parafilm^®^ and incubated at 20°C. Measurements were taken and growth rates determined as above. The kosmo‐/chaotropicity of the medium with the added solution was calculated from values in Cray *et al*. (2013b).

### *Bacillus* sp. JC12GB43:*F. coeruleum* interaction assays on potato tubers

A total of 1000 potato tubers (500 of cultivar Désirée and 500 of cv. Dundrod) were used to determine the outcomes of interactions between *Bacillus* sp. JC12GB43:*F. coeruleum in vivo*. Prior to infection of potato tubers, *F. coeruleum* was cultured on PDA for 17 days at 20°C. Petri plates containing this pathogen were then flooded with NaCl solution (0.8%, w/v), and the surface of the medium was immediately wiped using a sterile glass spreader in order to displace the macroconidia. The resulting spore suspension was then poured through a sterile funnel containing sterile glass wool to remove hyphal fragments. The concentration of macroconidia was determined using a haemocytometer, and the spore suspension adjusted to a concentration of 5 × 10^3^ macroconidia ml^−1^. Three litres of this suspension were added to 3 kg sterile soil, and mixed to produce a slurry. Potato‐tuber periderm was removed locally using a stainless steel‐wool file (20‐mm wide half‐round file; B&Q, Hampshire, UK) to create an area available for infection approximately 2‐cm wide, 4‐cm long and 0.5‐mm deep. The wounded tubers were placed immediately into polyethylene mesh bags (25 per bag, 20 bags of cultivar Désirée from potato grower T. Newell and 20 bags of cultivar Dundrod from Agri‐Food and Bioscience Institute, Newforge), dipped into the slurry containing *F. coeruleum* macroconidia and shaken for 2 min to ensure surface coverage with slurry, and then bags were distributed evenly, without contact with each other, on shelves and left to dry overnight in a temperature‐regulated glasshouse at 25°C. After 24 h, potato tubers were inoculated with *Bacillus* sp. JC12GB43 that had been cultured in NB (no added solute) (Treatment 1), NB+2 M glycerol (Treatment 2), or *Bacillus* sp. JC12GB43 that had been cultured in NB+2 M glycerol (Treatment 3) as described below. Control tubers had been inoculated with *F. coeruleum* slurry only; and the negative‐control tubers were not inoculated with biocontrol agent or *F. coeruleum* slurry. For the purposes of tuber inoculations, *Bacillus* sp. JC12GB43 had been cultured in 5‐L Erlenmeyer flasks containing NB (no added solute) and NB+2 M glycerol by inoculating (2 ml) taken from 16‐h cultures of *Bacillus* sp. JC12GB43 that had been subcultured in identical media, either NB or NB+2 M glycerol to an OD_600nm_ of 1. Flasks were then incubated (170 rpm; 30°C) for 24 h (NB; no added solute) or 72 h (NB+2 M glycerol). The OD_600nm_ of each *Bacillus* cultures was adjusted to 1.444 by the addition of appropriate medium (either NB or NB+2 M glycerol). Tuber inoculations were carried out by dipping the mesh bags containing infected tubers into 5 L of *Bacillus* sp. JC12GB43 culture in either NB or NB+2 M glycerol and shaking for 2 mins. Bags containing tubers were stored in five randomized block‐formations each containing a bag of each replicate, in a humidity‐regulated potato store (60% relative humidity; without light; at ambient temperature which varied between 8 and 18°C).

Tubers were checked regularly for signs of dry rot and, after 14 weeks (25/02/2014), there was sufficient rot to carry out an assessment. Assessments were made by halving the tubers through the infection site (if present) and – as a measure of severity – the percentage of the tuber infected with dry rot was recorded. As measurements were made by eye, bias was eliminated by labelling treatment bags with number only (so treatments were unknown). Data were analysed to show percentage of tubers with rot, mean area of rot in infected tubers only, and mean rot for total tubers; and were analysed using one‐way ANOVA and Tukey's *post hoc* analyses using GraphPad Prism 5.01 (Fig. 6).

## Supporting information


**Fig. S1.** Flow chart to illustrate the progression of work during the current study.
**Fig. S2.** Interactions between *F. sambucinum* and biocontrol agents on (A–C) NA and (D–F) PDA: (A and D) radial extension of *F. sambucinum* on the side adjacent to the biocontrol agent (dotted lines indicate the distance between *F. sambucinum* and biocontrol agent at the time of inoculation; (B and E) extent of colony of biocontrol agent beyond the initial zone‐of‐inoculation (over time), on the side adjacent to *F. sambucinum*; and (C and F) distance between *F. sambucinum* and biocontrol agent over time.
**Fig. S3.** Interactions between biocontrol agents and (A–C) *P. infestans* isolates 10LD3 or (D–F) 10D2_5 on CA: (A and D) radial extension of *P. infestans* on the side adjacent to the biocontrol agent (dotted lines indicate the distance between *P. infestans* and biocontrol agent at the time of inoculation; (C and F) extent of colony of biocontrol agent beyond the initial zone‐of‐inoculation (over time), on the side adjacent to *P. infestans*; and (C and F); distance between *P. infestans* and biocontrol agent over time.
**Fig. S4.** Interactions between *P. infestans* isolates 10LD3 (A–C) or 10D2_5 (D–F) and biocontrol agents on CA: (A and D) radial extension of *P. infestans* on the side adjacent to the biocontrol agent (dotted lines indicate the distance between *P. infestans* and biocontrol agent at the time of inoculation; (B and E); extent of colony of biocontrol agent beyond the initial zone‐of‐inoculation (over time), on the side adjacent to *P. infestans*; and (C and F); distance between *P. infestans* and biocontrol agent over time. *Phytophthora infestans* 10LD3 control (●), *Phytophthora infestans* 10D2_5 control (■), JC12GB51 (⨂), JC12GB50 (▲), JC12GB48 (▼), JC12GB47 (♢), JC12GB36 (×), JC12GB35 (+), JC12GB12 (*), JC12GB13 (▽), JC12GB12 (□), JC12GB7 (○), JC12GB6 (♦), JC12GB29 (⊠), JC12GB28 (a – c: I; d – f: I). Upon inoculation of CA, *P. infestans* 10LD3 and 10D2_5 were placed 25‐ and 16‐mm apart from the biocontrol agents respectively. Error bars indicate ± standard error.
**Fig. S5.** Interactions between *F. sambucinum* and biocontrol agents on NA+1.104 M NaCl: (A) adial extension of *F. sambucinum* on the side adjacent to the biocontrol agent (dotted lines indicate the distance between *F. sambucinum* and biocontrol agent at the time of inoculation); (B) extent of colony of biocontrol agent beyond the initial zone‐of‐inoculation (over time), on the side adjacent to *F. sambucinum*); (C) distance between *F. sambucinum* and biocontrol agent over time. *F. sambucinum* control (●), JC12GB189 (■), JC12GB191 (⨂), JC12GB196 (♢), JC12GB197 (□), JC12GB190 (○). Upon inoculation of NA+1.104 M NaCl, *F. sambucinum* and biocontrol agents were placed 13‐mm apart. Error bars indicate ± standard error.
**Fig. S6.** Radial growth rate of (A) *F. coeruleum*, (B) *F. sambucinum*, (C) *P. infestans* 10LD3 and (D) *P. infestans* 10D2_5 over a range of water activity values.
**Fig. S7.** Radial extension of *F. coeruleum* over a range of urea concentrations (0 mM: ●; 50 mM: ■; 100 mM: ▲; 150 mM: ○; 200 mM: □; 250 mM: ▵) and temperatures (5.2°C: A; 11.6°C: B; 13.4°C: C; 19.8°C: D; 23.2°C: E; 30.2°C: F).
**Fig. S8.** Radial growth rates of potato pathogens on various media at 20°C: (A) *F. coeruleum*, (B) *F. sambucinum*, (C) *P. Infestans* 10LD3 and (D) *P. infestans* 10D2_5. Error bars indicate + standard error.

**Table S1.** Key parameters and inhibition coefficients, obtained from interaction assays between biocontrol agents and *Fusarium coeruleum* ^a^.
**Table S2.** Key parameters and inhibition coefficients, obtained from interaction assays between biocontrol agents and *Fusarium sambucinum* ^a^.
**Table S3.** Key parameters and inhibition coefficients, obtained from interaction assays between biocontrol agents and *Phytophthora infestans* 10LD3^a^.
**Table S4.** Key parameters and inhibition coefficients, obtained from interaction assays between biocontrol agents and *Phytophthora infestans* 10D2_5^a^.
**Table S5.** Key parameters and inhibition coefficients, obtained from interaction assays between *Bacillus* sp. JC12GB43 and *Fusarium coeruleum* and on PDA supplemented with either glycerol or urea^a^.
**Table S6.** Colony development of *Fusarium coeruleum* on PDA supplemented with 132 mM urea in relation to exogenous addition of compatible solutes and other substances.

## Notes

1 Water activity, the mole fraction of water in the vapour phase, is numerically equivalent to the corresponding relative‐humidity value divided by 100. Although relative humidity is used to describe the mole fraction of water in the vapour phase, water activity is used for the liquid or solid phase. Water activity is a potent determinant for activity of biological systems (see Stevenson *et al*., 2015a,b).

2 This urea concentration was selected because it only inhibits growth rate of *Bacillus* sp. JC12GB43 by 75% relative to the control (Fig. 10A).
