# Supplementary material for: Biocontrol agents promote growth of potato pathogens, depending on environmental conditions
Source: Microb Biotechnol. 2016 Feb 16;9(3):330–54. doi: 10.1111/1751-7915.12349 (PMC4835571; doi:10.1111/1751-7915.12349)
Supplement: Supplementary file 1 — Fig. S1. Flow chart to illustrate the progression of work during the current study. Fig. S2. Interactions between F. sambucinum and biocontrol agents on (A–C) NA and (D–F) PDA: (A and D) radial extension of F. sambucinum on the side adjacent to the biocontrol agent (dotted lines indicate the distance between F. sambucinum and biocontrol agent at the time of inoculation; (B and E) extent of colony of biocontrol agent beyond the initial zone‐of‐inoculation (over time), on the side adjacent to F. sambucinum; and (C and F) distance between F. sambucinum and biocontrol agent over time. Fig. S3. Interactions between biocontrol agents and (A–C) P. infestans isolates 10LD3 or (D–F) 10D2_5 on CA: (A and D) radial extension of P. infestans on the side adjacent to the biocontrol agent (dotted lines indicate the distance between P. infestans and biocontrol agent at the time of inoculation; (C and F) extent of colony of biocontrol agent beyond the initial zone‐of‐inoculation (over time), on the side adjacent to P. infestans; and (C and F); distance between P. infestans and biocontrol agent over time. Fig. S4. Interactions between P. infestans isolates 10LD3 (A–C) or 10D2_5 (D–F) and biocontrol agents on CA: (A and D) radial extension of P. infestans on the side adjacent to the biocontrol agent (dotted lines indicate the distance between P. infestans and biocontrol agent at the time of inoculation; (B and E); extent of colony of biocontrol agent beyond the initial zone‐of‐inoculation (over time), on the side adjacent to P. infestans; and (C and F); distance between P. infestans and biocontrol agent over time. Phytophthora infestans 10LD3 control (●), Phytophthora infestans 10D2_5 control (■), JC12GB51 (⨂), JC12GB50 (▲), JC12GB48 (▼), JC12GB47 (♢), JC12GB36 (×), JC12GB35 (+), JC12GB12 (*), JC12GB13 (▽), JC12GB12 (□), JC12GB7 (○), JC12GB6 (♦), JC12GB29 (⊠), JC12GB28 (a – c: I; d – f: I). Upon inoculation of CA, P. infestans 10LD3 and 10D2_5 were placed 25‐ and 16‐mm apart from the [file MBT2-9-330-s001.pptx]

## Slide 1
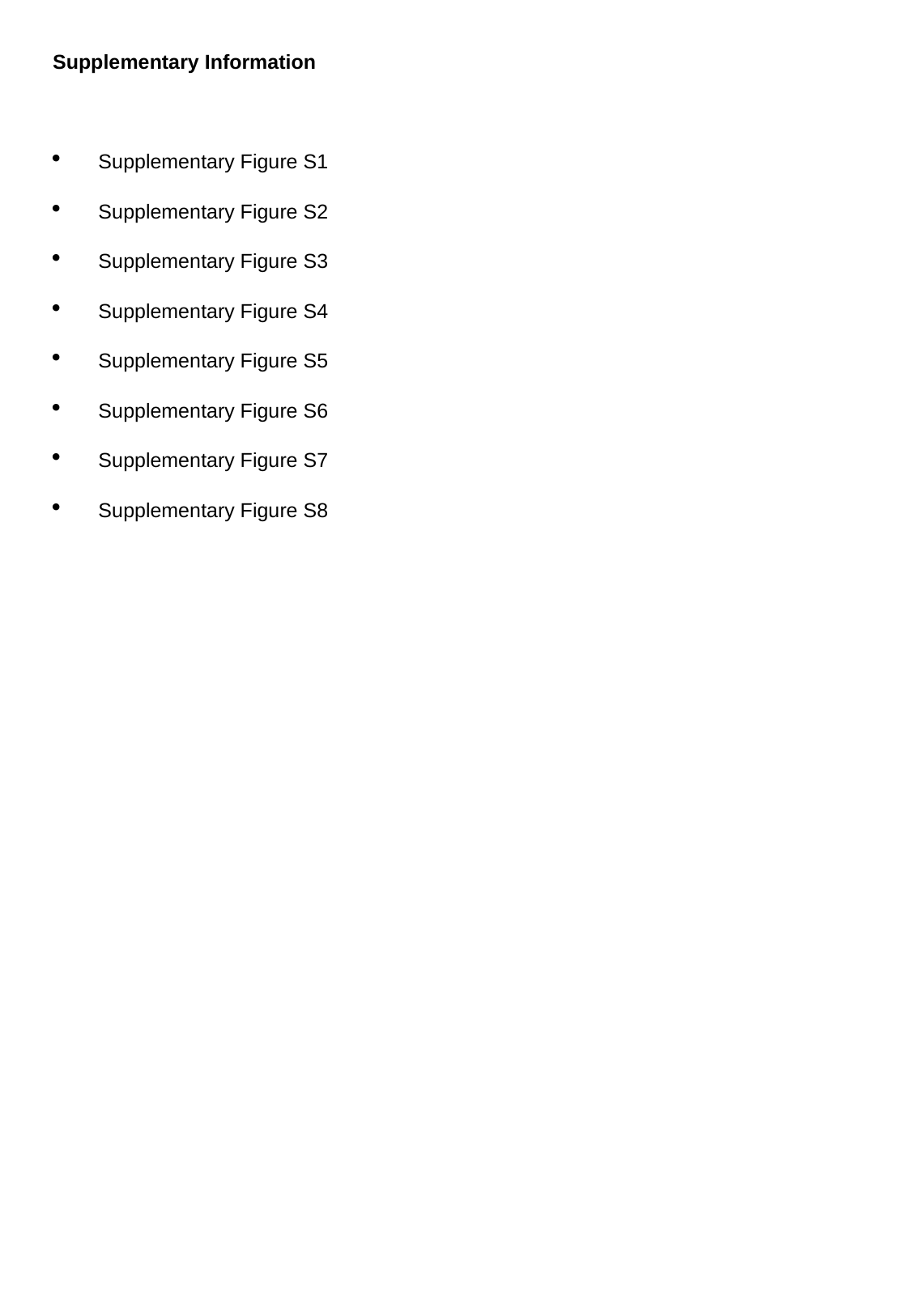

Supplementary Information
Supplementary Figure S1
Supplementary Figure S2
Supplementary Figure S3
Supplementary Figure S4
Supplementary Figure S5
Supplementary Figure S6
Supplementary Figure S7
Supplementary Figure S8

## Slide 2
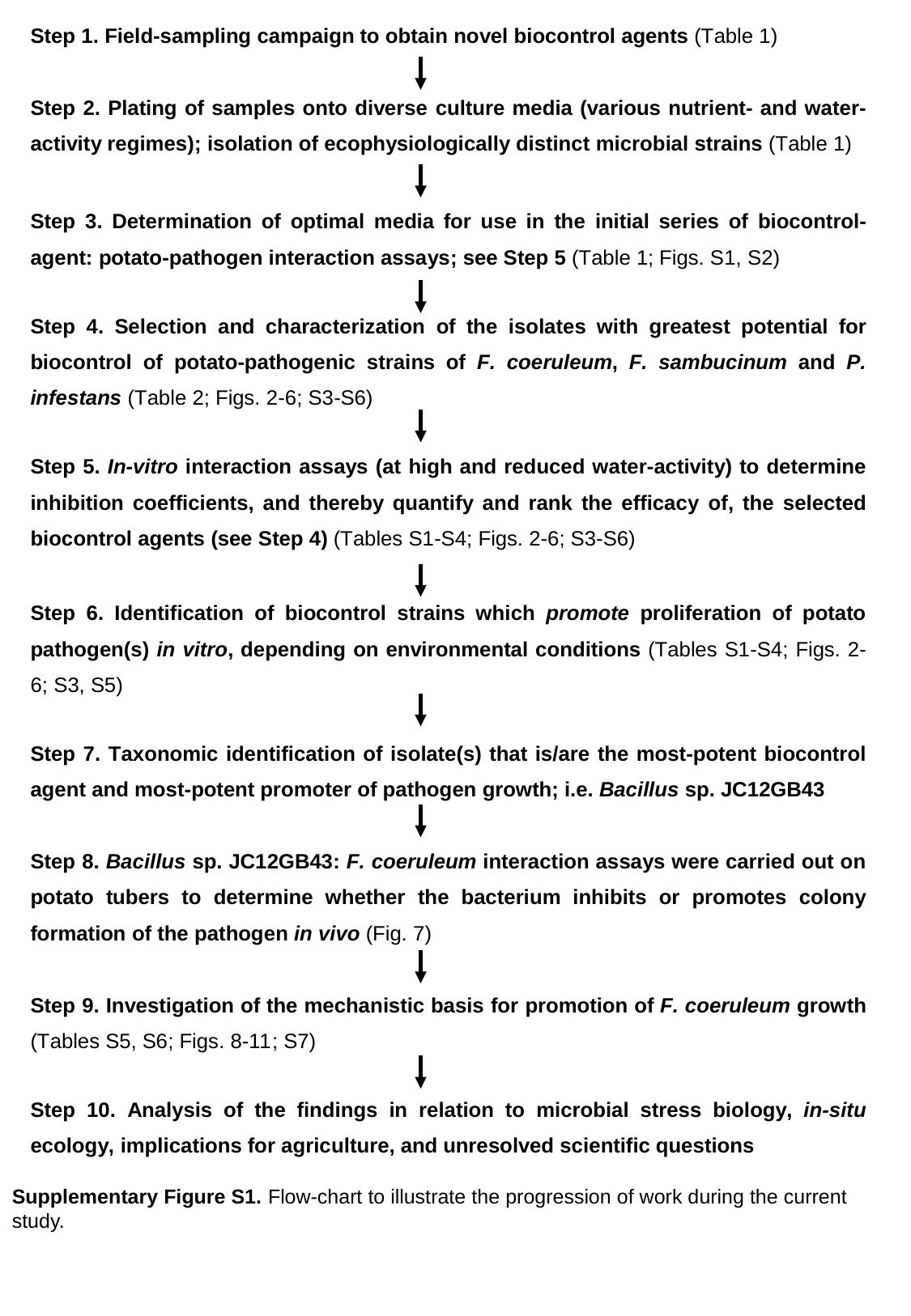

Supplementary Figure S1. Flow-chart to illustrate the progression of work during the current study.

## Slide 3
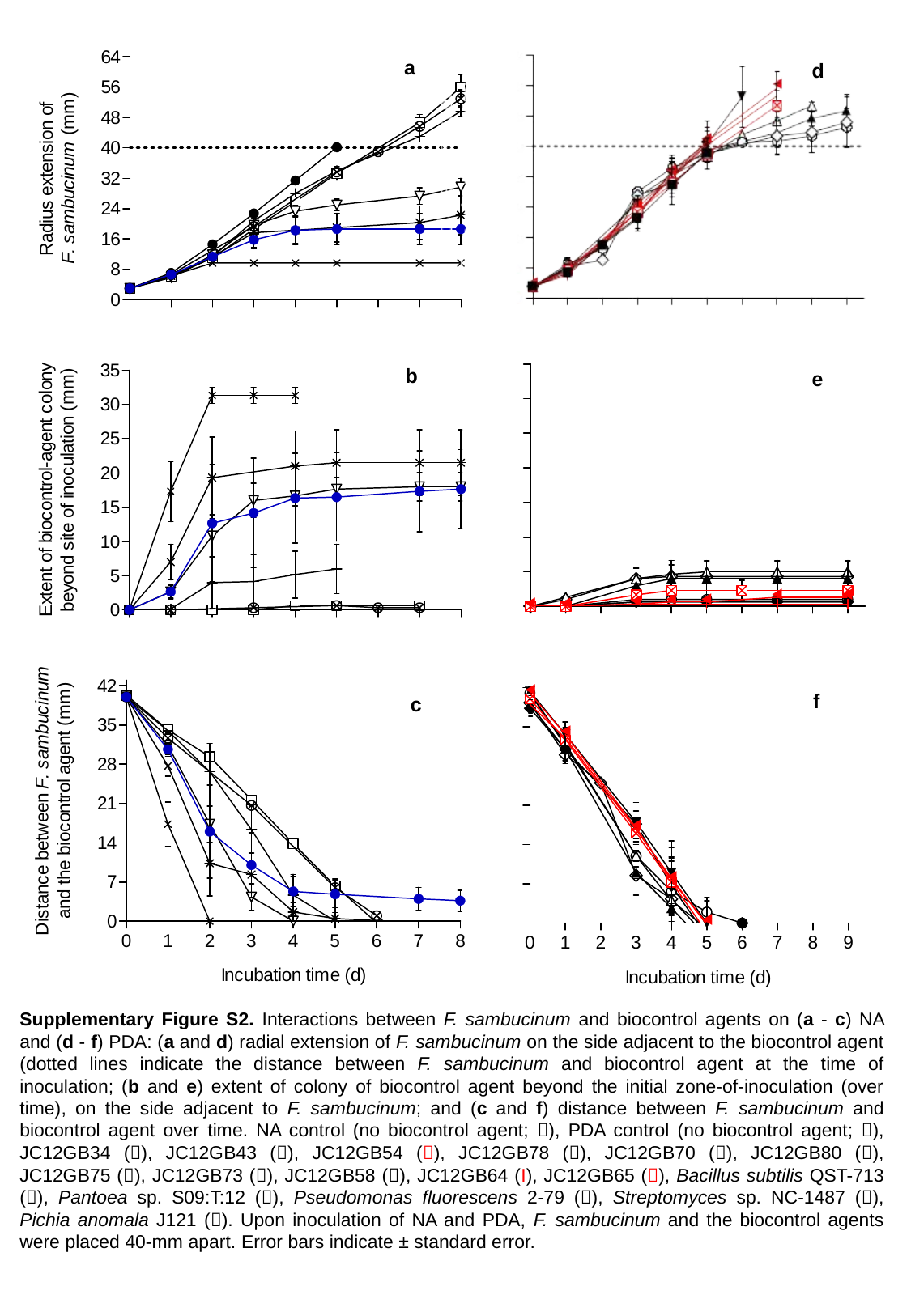

a
d
b
e
f
c
Supplementary Figure S2. Interactions between F. sambucinum and biocontrol agents on (a - c) NA and (d - f) PDA: (a and d) radial extension of F. sambucinum on the side adjacent to the biocontrol agent (dotted lines indicate the distance between F. sambucinum and biocontrol agent at the time of inoculation; (b and e) extent of colony of biocontrol agent beyond the initial zone-of-inoculation (over time), on the side adjacent to F. sambucinum; and (c and f) distance between F. sambucinum and biocontrol agent over time. NA control (no biocontrol agent; ), PDA control (no biocontrol agent; ), JC12GB34 (), JC12GB43 (), JC12GB54 (), JC12GB78 (), JC12GB70 (), JC12GB80 (), JC12GB75 (), JC12GB73 (), JC12GB58 (ρ), JC12GB64 (I), JC12GB65 (), Bacillus subtilis QST-713 (), Pantoea sp. S09:T:12 (), Pseudomonas fluorescens 2-79 (), Streptomyces sp. NC-1487 (), Pichia anomala J121 (). Upon inoculation of NA and PDA, F. sambucinum and the biocontrol agents were placed 40-mm apart. Error bars indicate ± standard error.

## Slide 4
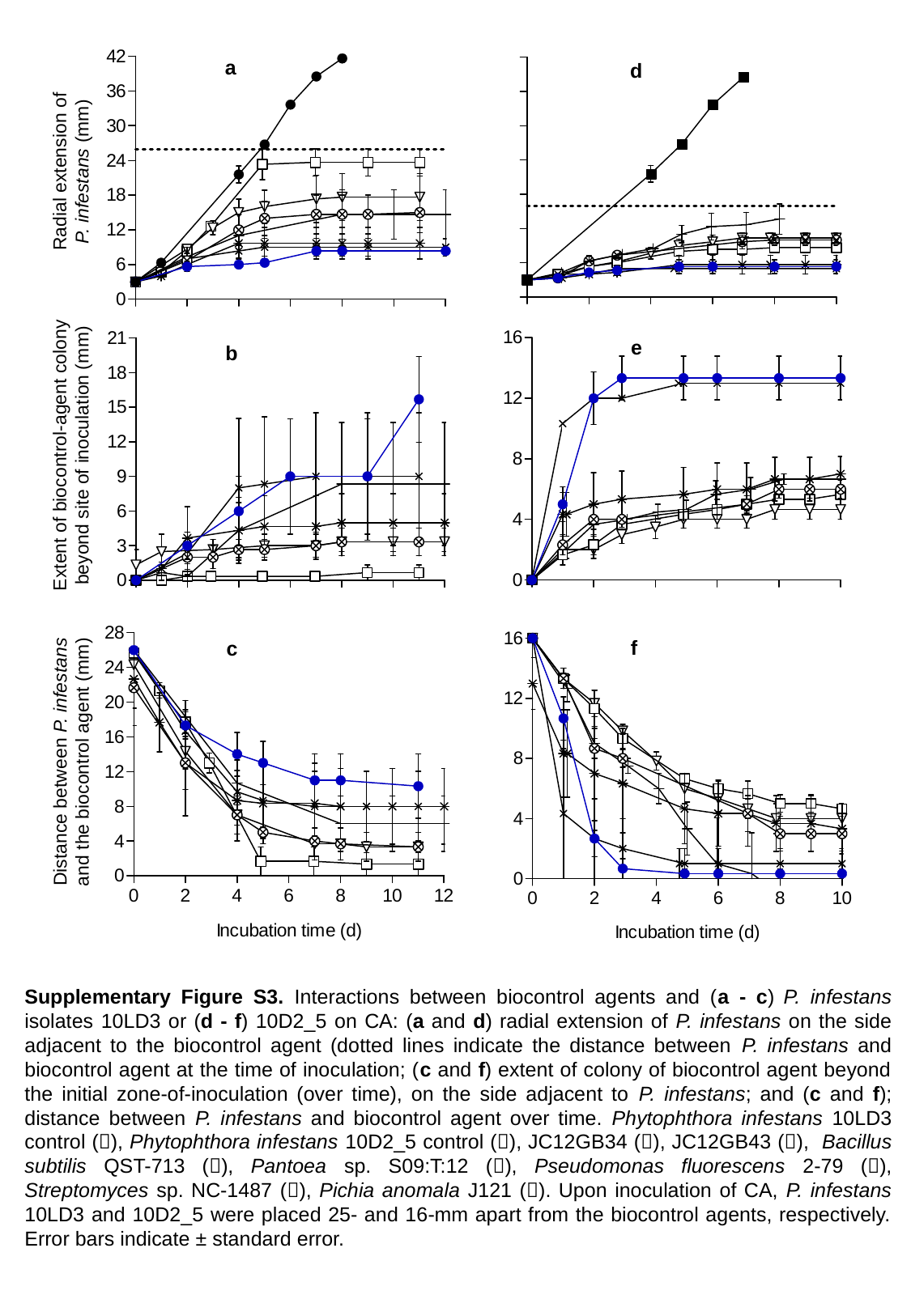

a
d
Radial extension of P. infestans (mm)
e
b
Extent of biocontrol-agent colony beyond site of inoculation (mm)
f
c
Distance between P. infestans and the biocontrol agent (mm)
Supplementary Figure S3. Interactions between biocontrol agents and (a - c) P. infestans isolates 10LD3 or (d - f) 10D2_5 on CA: (a and d) radial extension of P. infestans on the side adjacent to the biocontrol agent (dotted lines indicate the distance between P. infestans and biocontrol agent at the time of inoculation; (c and f) extent of colony of biocontrol agent beyond the initial zone-of-inoculation (over time), on the side adjacent to P. infestans; and (c and f); distance between P. infestans and biocontrol agent over time. Phytophthora infestans 10LD3 control (), Phytophthora infestans 10D2_5 control (), JC12GB34 (), JC12GB43 (), Bacillus subtilis QST-713 (), Pantoea sp. S09:T:12 (), Pseudomonas fluorescens 2-79 (), Streptomyces sp. NC-1487 (), Pichia anomala J121 (). Upon inoculation of CA, P. infestans 10LD3 and 10D2_5 were placed 25- and 16-mm apart from the biocontrol agents, respectively. Error bars indicate ± standard error.

## Slide 5
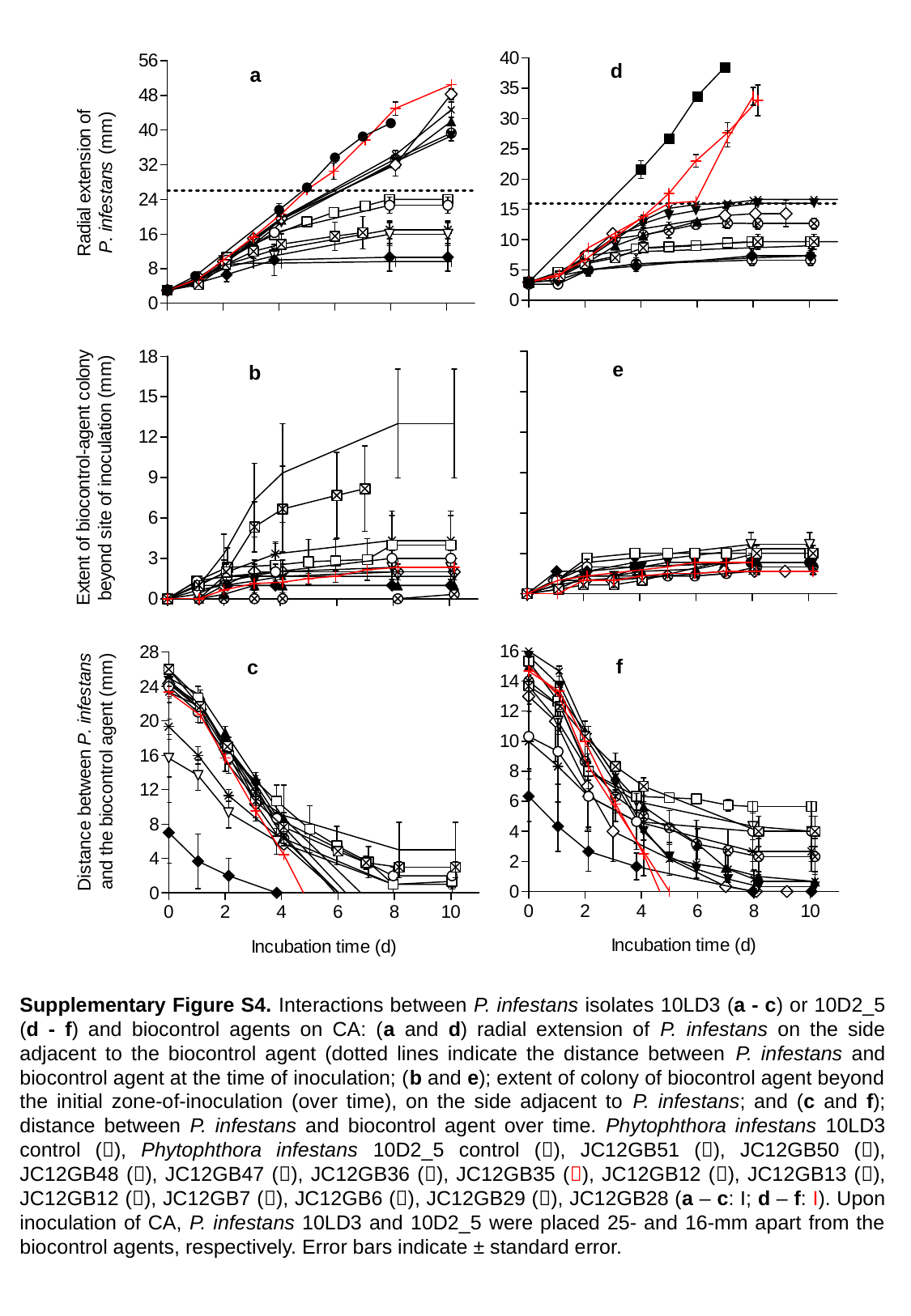

d
a
e
b
f
c
Supplementary Figure S4. Interactions between P. infestans isolates 10LD3 (a - c) or 10D2_5 (d - f) and biocontrol agents on CA: (a and d) radial extension of P. infestans on the side adjacent to the biocontrol agent (dotted lines indicate the distance between P. infestans and biocontrol agent at the time of inoculation; (b and e); extent of colony of biocontrol agent beyond the initial zone-of-inoculation (over time), on the side adjacent to P. infestans; and (c and f); distance between P. infestans and biocontrol agent over time. Phytophthora infestans 10LD3 control (), Phytophthora infestans 10D2_5 control (), JC12GB51 (), JC12GB50 (), JC12GB48 (), JC12GB47 (), JC12GB36 (), JC12GB35 (), JC12GB12 (), JC12GB13 (), JC12GB12 (), JC12GB7 (), JC12GB6 (), JC12GB29 (), JC12GB28 (a – c: I; d – f: I). Upon inoculation of CA, P. infestans 10LD3 and 10D2_5 were placed 25- and 16-mm apart from the biocontrol agents, respectively. Error bars indicate ± standard error.

## Slide 6
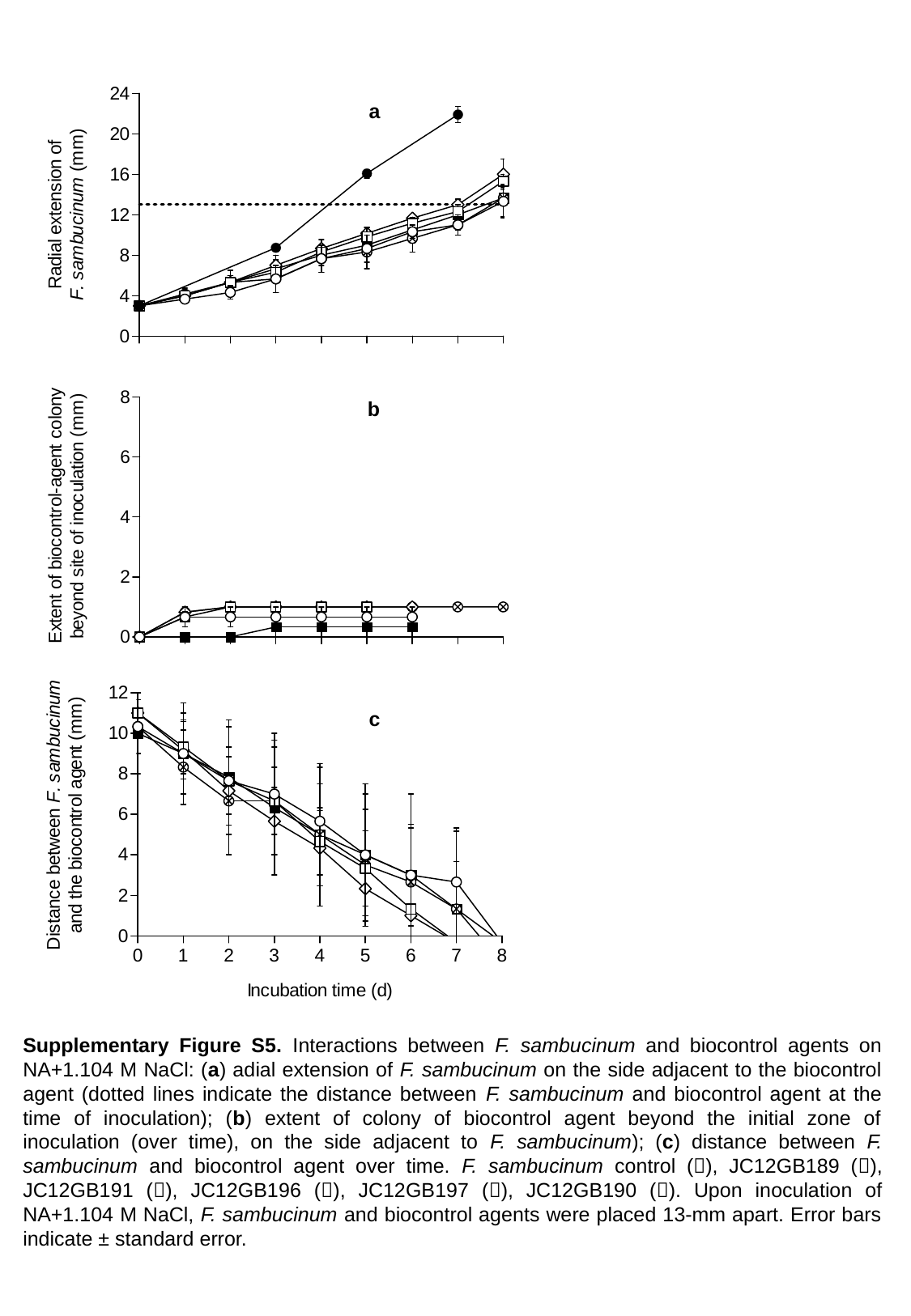

a
b
c
Supplementary Figure S5. Interactions between F. sambucinum and biocontrol agents on NA+1.104 M NaCl: (a) adial extension of F. sambucinum on the side adjacent to the biocontrol agent (dotted lines indicate the distance between F. sambucinum and biocontrol agent at the time of inoculation); (b) extent of colony of biocontrol agent beyond the initial zone of inoculation (over time), on the side adjacent to F. sambucinum); (c) distance between F. sambucinum and biocontrol agent over time. F. sambucinum control (), JC12GB189 (), JC12GB191 (), JC12GB196 (), JC12GB197 (), JC12GB190 (). Upon inoculation of NA+1.104 M NaCl, F. sambucinum and biocontrol agents were placed 13-mm apart. Error bars indicate ± standard error.

## Slide 7
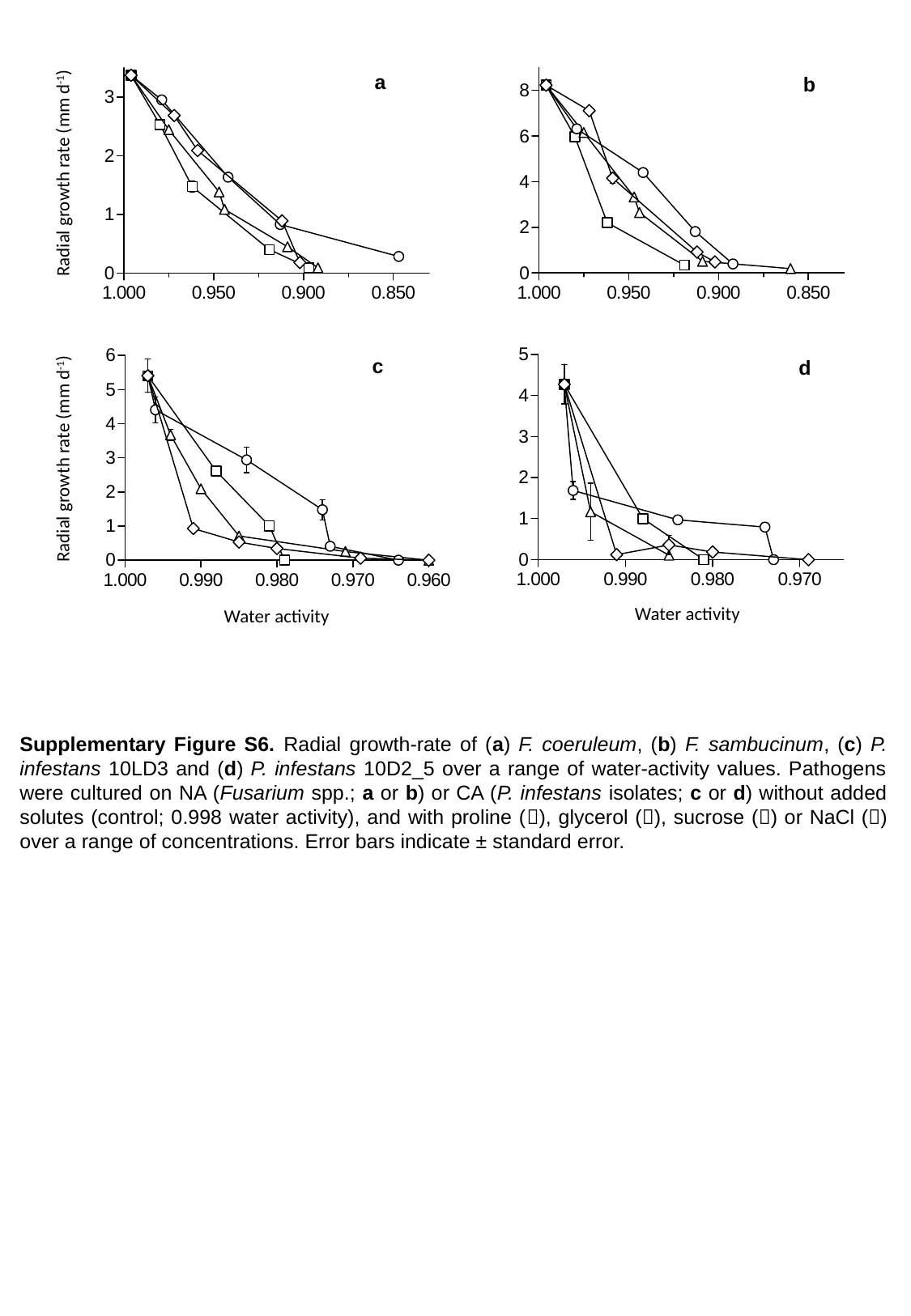

a
b
Radial growth rate (mm d-1)
c
d
Radial growth rate (mm d-1)
Water activity
Water activity
Supplementary Figure S6. Radial growth-rate of (a) F. coeruleum, (b) F. sambucinum, (c) P. infestans 10LD3 and (d) P. infestans 10D2_5 over a range of water-activity values. Pathogens were cultured on NA (Fusarium spp.; a or b) or CA (P. infestans isolates; c or d) without added solutes (control; 0.998 water activity), and with proline (ρ), glycerol (), sucrose () or NaCl () over a range of concentrations. Error bars indicate ± standard error.

## Slide 8
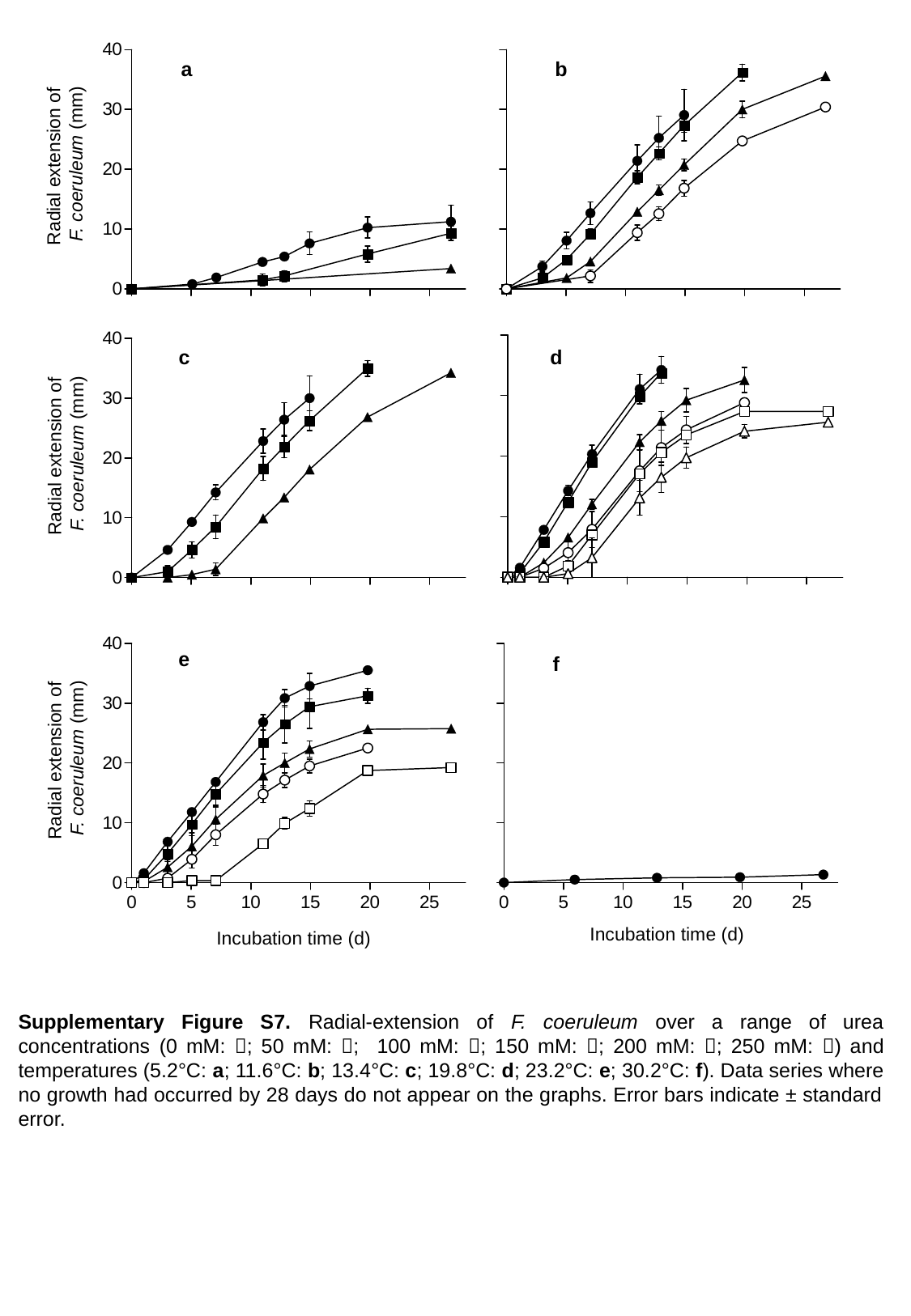

a
b
Radial extension of
F. coeruleum (mm)
c
d
Radial extension of
F. coeruleum (mm)
e
f
Radial extension of
F. coeruleum (mm)
Incubation time (d)
Incubation time (d)
Supplementary Figure S7. Radial-extension of F. coeruleum over a range of urea concentrations (0 mM: ; 50 mM: ; 100 mM: ; 150 mM: ; 200 mM: ; 250 mM: ) and temperatures (5.2°C: a; 11.6°C: b; 13.4°C: c; 19.8°C: d; 23.2°C: e; 30.2°C: f). Data series where no growth had occurred by 28 days do not appear on the graphs. Error bars indicate ± standard error.

## Slide 9
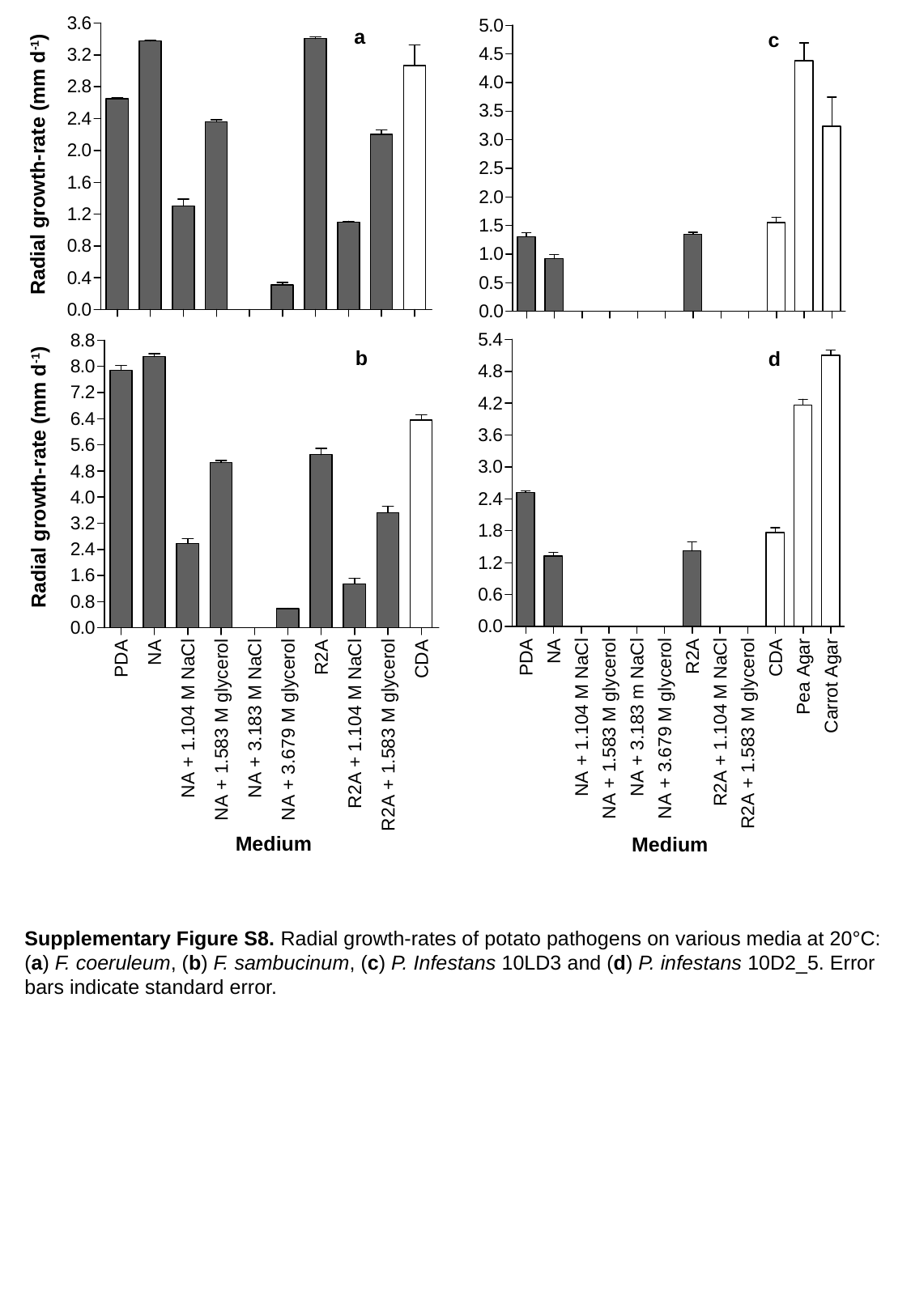

a
c
Radial growth-rate (mm d-1)
b
d
Radial growth-rate (mm d-1)
Medium
Medium
Supplementary Figure S8. Radial growth-rates of potato pathogens on various media at 20°C: (a) F. coeruleum, (b) F. sambucinum, (c) P. Infestans 10LD3 and (d) P. infestans 10D2_5. Error bars indicate standard error.
