# Supplementary material for: Biocontrol agents promote growth of potato pathogens, depending on environmental conditions
Source: Microb Biotechnol. 2016 Feb 16;9(3):330–54. doi: 10.1111/1751-7915.12349 (PMC4835571; doi:10.1111/1751-7915.12349)
Supplement: Supplementary file 2 — Table S1. Key parameters and inhibition coefficients, obtained from interaction assays between biocontrol agents and Fusarium coeruleum a. Table S2. Key parameters and inhibition coefficients, obtained from interaction assays between biocontrol agents and Fusarium sambucinum a. Table S3. Key parameters and inhibition coefficients, obtained from interaction assays between biocontrol agents and Phytophthora infestans 10LD3a. Table S4. Key parameters and inhibition coefficients, obtained from interaction assays between biocontrol agents and Phytophthora infestans 10D2_5a. Table S5. Key parameters and inhibition coefficients, obtained from interaction assays between Bacillus sp. JC12GB43 and Fusarium coeruleum and on PDA supplemented with either glycerol or ureaa. Table S6. Colony development of Fusarium coeruleum on PDA supplemented with 132 mM urea in relation to exogenous addition of compatible solutes and other substances. [file MBT2-9-330-s002.docx]

**Supporting Information**

- Table S1
- Table S2
- Table S3
- Table S4
- Table S5
- Table S6
- Supporting references

**Table S1.** Key parameters and inhibition coefficients, obtained from interaction assays between biocontrol agents and *Fusarium coeruleum*^a^

| Potential biocontrol agents | Culture medium^b^ | *F.* *coeruleum* growth rate (mm day^-1^); [*A*]^c^ | Growth rate [*A*] as a percentage of control; [*B*] | Growth of *F. coeruleum* as a percentage of the distance between sites of inoculation; [*C*] | Time until contact between *F. coeruleum* and biocontrol agent (days) | *F. coeruleum* growth rate in zone of mixed culture (mm day^-1^); [D]^d^ | Growth rate [*D*] as percentage of control; [*E*] | Inhibition coefficient^e^ |
| --- | --- | --- | --- | --- | --- | --- | --- | --- |
| JC12GB43 | NA | 1.00 ± 0.00 | 30.5 ± 0.00 | 6.68 ± 0.15 | 3.00 ± 0.00 | 0.00 ± 0.00 | 0.00 ± 0.00 | **84.2** ± 0.77(a) |
| *Pseudomonas fluorescens* 2-79 | NA | 1.00 ± 0.00 | 30.5 ± 0.00 | 8.33 ± 0.15 | 2.33 ± 1.15 | 0.50 ± 0.00 | 15.3 ± 0.00 | **80.3** ± 0.77(a) |
| *Bacillus subtilis* QST 713 | NA | 1.69 ± 0.60 | 51.6 ± 18.3 | 10 ± 4.33 | No overlap between cultures | No overlap between cultures | No overlap between cultures | **74.0** ± 9.63(ab) |
| *Streptomyces* sp. NC-1487 | NA | 1.12 ± 0.21 | 34.2 ± 6.30 | 13.3 ± 5.20 | 2.76 ± 0.21 | 1.00 ± 0.00 | 30.5 ± 0.00 | **73.1** ± 5.29(ab) |
| *Pichia anomala* J121 | NA | 2.87 ± 0.02 | 87.7 ± 0.52 | 64.3 ± 1.45 | No overlap between cultures | No overlap between cultures | No overlap between cultures | **30.7** ± 0.98(cd) |
| JC12GB34 | NA | 2.93 ± 0.27 | 89.3 ± 0.11 | > 100 ± 0.00 | 10.9 ± 1.13 | 1.12 ± 0.21 | 34.2 ± 6.30 | **17.4** ± 1.30(cde) |
| JC12GB12 | NA+glyc | 2.04 ± 0.77 | 86.6 ± 32.8 | > 100 ± 0.00 | 3.07 ± 0.43 | 1.21 ± 0.46 | 51.3 ± 19.3 | **15.1** ± 17.0(cde) |
| *Pantoea* sp. S09:T:12 | NA | 3.02 ± 0.11 | 92.3 ± 3.46 | > 100 ± 0.00 | 9.09 ± 0.18 | 1.36 ± 1.12 | 41.4 ± 34.1 | **14.8** ± 8.20(cde) |
| JC12GB7 | NA+glyc | 2.18 ± 0.29 | 92.5 ± 12.3 | > 100 ± 0.00 | 3.07 ± 0.43 | 1.36 ± 0.58 | 57.5 ± 24.4 | **11.5** ± 9.80(def) |
| JC12GB70 | PDA | 2.21 ± 0.21 | 83.5 ± 8.02 | > 100 ± 0.00 | 5.19 ± 1.75 | 2.55 ± 0.33 | 96.3 ± 12.5 | **7.34** ± 5.71(def) |
| JC12GB14 | NA+glyc | 2.18 ± 0.20 | 92.2 ± 8.63 | > 100 ± 0.00 | 3.61 ± 0.40 | 1.88 ± 0.77 | 79.6 ± 32.4 | **7.18** ± 9.94(def) |
| JC12GB73 | PDA | 2.83 ± 1.04 | 107 ± 39.3 | > 100 ± 0.00 | 4.89 ± 0.19 | 1.50 ± 0.50 | 56.7 ± 18.9 | **5.86** ± 19.5(def) |
| JC12GB13 | NA+glyc | 2.54 ± 0.45 | 108 ± 19.2 | > 100 ± 0.00 | 3.47 ± 0.72 | 1.52 ± 1.17 | 64.2 ± 49.5 | **4.04** ± 17.6(def) |
| JC12GB75 | PDA | 2.75 ± 0.16 | 104 ± 5.90 | > 100 ± 0.00 | 4.85 ± 1.19 | 2.06 ± 0.89 | 77.7 ± 33.8 | **2.92** ± 9.11(def) |
| JC12GB6 | NA+glyc | 2.56 ± 0.07 | 109 ± 3.06 | > 100 ± 0.00 | 5.34 ± 0.58 | 2.06 ± 0.93 | 87.2 ± 39.3 | **-0.86** ± 9.09(def) |
| JC12GB189 | NA+NaCl | 1.26 ± 0.08 | 96.7 ± 6.17 | > 100 ± 0.00 | 5.81 ± 2.14 | 1.59 ± 0.22 | 122 ± 16.84 | **-3.16** ± 5.84(ef) |
| JC12GB61 | PDA | 3.20 ± 0.23 | 121 ± 8.80 | > 100 ± 0.00 | 3.89 ± 0.82 | 2.34 ± 0.79 | 88.5 ± 29.9 | **-6.03** ± 9.49(ef) |
| JC12GB78 | PDA | 3.33 ± 0.58 | 126 ± 21.8 | > 100 ± 0.00 | 5.11 ± 1.02 | 2.17 ± 1.61 | 81.8 ± 60.7 | **-6.73** ± 20.9(ef) |
| JC12GB196 | NA+NaCl | 1.34 ± 0.30 | 103 ± 23.1 | > 100 ± 0.00 | 4.58 ± 2.47 | 1.65 ± 0.39 | 127 ± 29.7 | **-6.59** ± 15.2(ef) |
| JC12GB191 | NA+NaCl | 1.35 ± 0.06 | 104 ± 4.93 | > 100 ± 0.00 | 6.01 ± 1.82 | 1.76 ± 0.33 | 135 ± 25.6 | **-8.57** ± 7.09(ef) |
| JC12GB197 | NA+NaCl | 1.37 ± 0.13 | 106 ± 9.90 | > 100 ± 0.00 | 4.82 ± 2.71 | 1.77 ± 0.30 | 136 ± 22.8 | **-9.42** ± 8.51(ef) |
| JC12GB54 | PDA | 3.25 ± 0.22 | 123 ± 8.20 | > 100 ± 0.00 | 4.21 ± 1.12 | 4.33 ± 1.17 | 163 ± 44.3 | **-21.9** ± 12.1(f) |

a. All values are means of three independent replicates ± standard deviation.

b. NA + glyc = NA+1.583 M glycerol; NA + NaCl = NA+1.104 M NaCl

c. These values, for maximum growth-rate, were calculated using measurements of colony radius that were made only on the side of the colony adjacent to the potential biocontrol agent. Upon inoculation of NA+NaCl, NA+glycerol, PDA and NA, *F. coeruleum* and potential biological control agent were placed 7-, 13-, 13- and 40-mm apart, respectively.

d. These values indicate maximum growth-rate within zone of mixed culture.

e. Inhibition coefficients were calculated by the following equation: (100 − growth-rate *B*) × 0.4 + (100 − growth *C*) × 0.4 + (100 − growth-rate *E*) × 0.2; see Cray *et al*., (2015); values which do not include the same letter(s) in brackets are significantly different (*P* < 0.05). Values in red indicate promotion of pathogen growth.

**Table S2.** Key parameters and inhibition coefficients, obtained from interaction assays between biocontrol agents and *Fusarium sambucinum*^a^

| Potential biocontrol agents | Culture medium^b^ | *F.* *sambucinum* growth rate (mm day^-1^); [*A*]^c^ | Growth rate [*A*] as a percentage of control; [*B*] | Growth of *F. sambucinum* as a percentage of the distance between sites of inoculation; [*C*] | Time until contact between *F. sambucinum* and biocontrol agent (days) | *F. sambucinum* growth rate in zone of mixed culture (mm day^-1^); [D]^d^ | Growth rate [*D*] as percentage of control; [*E*] | Inhibition coefficient^e^ |
| --- | --- | --- | --- | --- | --- | --- | --- | --- |
| JC12GB43 | NA | 2.90 ± 0.61 | 35.0 ± 7.32 | 20.8 ± 2.88 | 2.09 ± 0.67 | 0.00 ± 0.00 | 0.00 ± 0.00 | **77.7** ± 4.08(a) |
| *Bacillus subtilis* QST 713 | NA | 3.58 ± 0.14 | 43.2 ± 1.74 | 16.7 ± 1.90 | 2.00 ± 0.00 | 0.00 ± 0.00 | 0.00 ± 0.00 | **76.1** ± 1.46(a) |
| *Pseudomonas fluorescens* 2-79 | NA | 5.63 ± 1.43 | 67.9 ± 17.2 | 48.3 ± 22.4 | 4.40 ± 2.70 | 0.93 ± 0.12 | 11.2 ± 1.39 | **51.3** ± 16.1(abc) |
| *Streptomyces* sp. NC-1487 | NA | 6.38 ± 0.46 | 76.9 ± 5.60 | 66.8 ± 10.1 | 3.67 ± 0.58 | 1.31 ± 0.86 | 15.8 ± 10.4 | **39.4** ± 8.36(bcd) |
| JC12GB189 | NA+NaCl | 1.19 ± 0.19 | 45.7 ± 7.41 | > 100 ± 0.00 | 5.25 ± 1.06 | 1.55 ± 0.60 | 45.7 ± 7.41 | **32.6** ± 4.44(bcde) |
| JC12GB191 | NA+NaCl | 1.28 ± 0.34 | 49.1 ± 13.0 | > 100 ± 0.00 | 8.90 ± 3.88 | 1.25 ± 0.57 | 48.0 ± 21.8 | **30.8** ± 9.56(bcdef) |
| JC12GB196 | NA+NaCl | 1.61 ± 0.28 | 62.0 ± 10.8 | > 100 ± 0.00 | 7.95 ± 2.66 | 1.56 ± 0.69 | 59.9 ± 26.6 | **23.2** ± 9.62(cdefg) |
| JC12GB197 | NA+NaCl | 1.65 ± 0.24 | 63.4 ± 9.07 | > 100 ± 0.00 | 7.67 ± 2.24 | 1.53 ± 0.33 | 58.8 ± 12.7 | **22.9** ± 6.17(cdefg) |
| JC12GB190 | NA+NaCl | 1.57 ± 0.51 | 60.3 ± 19.5 | > 100 ± 0.00 | 10.3 ± 4.98 | 2.23 ± 0.94 | 85.9 ± 36.0 | **18.7** ± 15.0(defg) |
| JC12GB73 | PDA | 7.40 ± 1.29 | 93.7 ± 16.3 | > 100 ± 0.00 | 5.92 ± 2.70 | 2.83 ± 2.47 | 35.9 ± 31.2 | **15.3** ± 12.8(defg) |
| JC12GB80 | PDA | 8.45 ± 0.65 | 107 ± 8.20 | > 100 ± 0.00 | 6.67 ± 0.58 | 1.17 ± 2.02 | 14.8 ± 25.6 | **14.3** ± 8.40(defg) |
| *Pantoea* sp. S09:T:12 | NA | 7.60 ± 0.74 | 91.6 ± 8.87 | > 100 ± 0.00 | 5.00 ± 1.00 | 4.33 ± 1.15 | 52.2 ± 13.9 | **12.9** ± 6.33(defg) |
| JC12GB34 | NA | 6.74 ± 0.44 | 81.2 ± 5.31 | > 100 ± 0.00 | 6.16 ± 0.17 | 6.67 ± 0.58 | 80.3 ± 6.96 | **11.5** ± 3.51(defg) |
| JC12GB58 | PDA | 8.26 ± 0.39 | 104 ± 4.95 | > 100 ± 0.00 | 4.64 ± 1.10 | 2.69 ± 0.60 | 34.1 ± 7.62 | **11.3** ± 3.51(defg) |
| *Pichia anomala* J121 | NA | 7.28 ± 1.01 | 87.8 ± 12.2 | > 100 ± 0.00 | 5.98 ± 0.39 | 6.67 ± 0.29 | 80.3 ± 3.48 | **8.84** ± 5.56(defg) |
| JC12GB70 | PDA | 7.53 ± 1.45 | 95.3 ± 18.3 | > 100 ± 0.00 | 5.34 ± 1.55 | 6.00 ± 2.35 | 75.9 ± 29.7 | **6.70** ± 13.3(efg) |
| JC12GB78 | PDA | 8.50 ± 0.50 | 108 ± 6.33 | > 100 ± 0.00 | 4.90 ± 1.15 | 4.17 ± 2.36 | 52.7 ± 29.9 | **6.41** ± 8.51(efg) |
| JC12GB75 | PDA | 8.06 ± 1.45 | 102 ± 18.3 | > 100 ± 0.00 | 5.24 ± 1.23 | 6.55 ± 2.84 | 82.9 ± 35.9 | **2.64** ± 14.5(fg) |
| JC12GB54 | PDA | 8.52 ± 0.63 | 108 ± 7.95 | > 100 ± 0.00 | 5.28 ± 1.47 | 6.71 ± 1.23 | 85.0 ± 15.5 | **-0.13** ± 6.29(g) |
| JC12GB64 | PDA | 9.07 ± 1.39 | 115 ± 17.6 | > 100 ± 0.00 | 5.16 ± 0.75 | 6.29 ± 1.69 | 79.6 ± 18.2 | **-1.83** ± 10.7(g) |
| JC12GB65 | PDA | 9.30 ± 0.63 | 117 ± 8.03 | > 100 ± 0.00 | 5.16 ± 0.94 | 6.83 ± 1.50 | 86.4 ± 19.0 | **-4.37** ± 7.00(g) |

a. All values are means of three independent replicates ± standard deviation.

b. NA+NaCl = NA+1.104 M NaCl.

c. These values, for maximum growth-rate, were calculated using measurements of colony radius that were made only on the side of the colony adjacent to the potential biocontrol agent. Upon inoculation of NA+NaCl, PDA and NA, *F. sambucinum* and potential biological control agent were placed 13-, 40- and 40-mm apart, respectively.

d. These values indicate maximum growth-rate within zone of mixed culture.

e. Inhibition coefficients were calculated by the following equation: (100 − growth-rate *B*) × 0.4 + (100 − growth *C*) × 0.4 + (100 − growth-rate *E*) × 0.2; see Cray *et al*., (2015); values which do not include the same letter(s) in brackets are significantly different (*P* < 0.05). Values in red indicate promotion of pathogen growth.

**Table S3.** Key parameters and inhibition coefficients, obtained from interaction assays between biocontrol agents and *Phytophthora infestans* 10LD3 ^a^

| Potential biocontrol agents | *P. infestans* 10LD3 growth rate (mm day^-1^); [*A*]^b^ | Growth rate [*A*] as a percentage of control; [*B*] | Growth of *P. infestans* 10LD3 as a percentage of the distance between sites of inoculation; [*C*] | Time until contact between *P. infestans* 10LD3 and biocontrol agent (days) | *P. infestans* 10LD3 growth rate in zone of mixed culture (mm day^-1^); [D]^c^ | Growth rate [*D*] as percentage of control; [*E*] | Inhibition coefficient^d^ |
| --- | --- | --- | --- | --- | --- | --- | --- |
| JC12GB43 | 1.33 ± 1.15 | 26.1 ± 22.6 | 6.42 ± 11.1 | < 1.00 ± 0.00 | 0.00 ± 0.00 | 0.00 ± 0.00 | **87.0** ± 13.5(a) |
| JC12GB6 | 2.26 ± 1.64 | 44.4 ± 32.1 | 29.5 ± 21.2 | 1.96 ± 1.55 | 0.00 ± 0.00 | 0.00 ± 0.00 | **70.4** ± 21.3(abc) |
| *Bacillus subtilis QST-713* | 3.00 ± 1.73 | 58.8 ± 34.0 | 23.1 ± 10.2 | No overlap between cultures | No overlap between cultures | 0.00 ± 0.00 | **67.2** ± 17.7(abcd) |
| *Pseudomonas fluorescens* 2-79 | 3.33 ± 1.53 | 65.4 ± 30.0 | 25.7 ± 18.2 | No overlap between cultures | No overlap between cultures | 0.00 ± 0.00 | **63.6** ± 19.3(abcde) |
| JC12GB28 | 4.19 ± 0.66 | 82.2 ± 12.9 | 25.7 ± 5.88 | No overlap between cultures | No overlap between cultures | 0.00 ± 0.00 | **56.9** ± 7.52(abcdef) |
| JC12GB34 | 3.17 ± 1.04 | 62.1 ± 20.4 | 46.2 ± 23.4 | No overlap between cultures | No overlap between cultures | 0.00 ± 0.00 | **56.7** ± 17.5(abcdef) |
| JC12GB13 | 3.08 ± 0.53 | 60.3 ± 10.5 | 50.0 ± 17.6 | No overlap between cultures | No overlap between cultures | 0.00 ± 0.00 | **55.9** ± 11.2(abcdef) |
| JC12GB14 | 3.38 ± 0.53 | 66.4 ± 10.5 | 53.8 ± 13.9 | No overlap between cultures | No overlap between cultures | 0.00 ± 0.00 | **51.9** ± 9.73(abcdefg) |
| *Pantoea* sp. S09:T:12 | 4.25 ± 0.66 | 83.4 ± 12.9 | 47.3 ± 25.6 | No overlap between cultures | No overlap between cultures | 0.00 ± 0.00 | **47.7** ± 15.4(bcdefgh) |
| *Streptomyces sp. NC-1487* | 4.33 ± 1.15 | 85.0 ± 22.6 | 56.5 ± 27.0 | No overlap between cultures | No overlap between cultures | 0.00 ± 0.00 | **43.5** ± 19.9(bcdefghi) |
| JC12GB29 | 4.83 ± 0.43 | 94.8 ± 8.49 | 51.2 ± 27.8 | No overlap between cultures | No overlap between cultures | 0.00 ± 0.00 | **41.6** ± 14.5(cdefghij) |
| JC12GB7 | 4.92 ± 0.53 | 96.5 ± 10.5 | 75.8 ± 12.3 | No overlap between cultures | No overlap between cultures | 0.00 ± 0.00 | **31.3** ± 9.13(defghijk) |
| JC12GB12 | 5.29 ± 0.43 | 104 ± 8.36 | 80.8 ± 3.85 | No overlap between cultures | No overlap between cultures | 0.00 ± 0.00 | **26.2** ± 4.88(efghijk) |
| *Pichia anomala* J121 | 6.32 ± 0.79 | 124 ± 15.5 | 79.6 ± 15.5 | No overlap between cultures | No overlap between cultures | 0.00 ± 0.00 | **18.7** ± 12.4(fghijk) |
| JC12GB48 | 4.24 ± 0.19 | 83.1 ± 3.69 | > 100 ± 0.00 | 5.90 ± 0.23 | 3.00 ± 0.50 | 58.8 ± 9.80 | **15.0** ± 3.44(ghijk) |
| JC12GB51 | 4.90 ± 0.40 | 96.0 ± 7.91 | > 100 ± 0.00 | 5.32 ± 0.38 | 3.00 ± 0.50 | 58.8 ± 9.80 | **9.84** ± 5.12(hijk) |
| JC12GB47 | 4.66 ± 0.11 | 91.3 ± 2.14 | > 100 ± 0.00 | 5.12 ± 0.20 | 4.24 ± 0.19 | 83.1 ± 3.69 | **6.84** ± 1.59(ijk) |
| JC12GB50 | 4.86 ± 0.23 | 95.2 ± 4.61 | > 100 ± 0.00 | 5.85 ± 0.22 | 4.83 ± 1.53 | 94.8 ± 30.0 | **2.95** ± 7.83(jk) |
| JC12GB36 | 4.96 ± 0.21 | 97.2 ± 4.15 | > 100 ± 0.00 | 5.46 ± 0.30 | 5.17 ± 1.15 | 101 ± 22.6 | **0.85** ± 6.19(k) |
| JC12GB35 | 5.30 ± 0.03 | 104 ± 0.62 | > 100 ± 0.00 | 4.86 ± 0.16 | 5.75 ± 3.55 | 113 ± 69.6 | **-4.09** ± 14.2(k) |

a. All assays were conducted on CA and values are means of three independent replicates ± standard deviation.

b. These values, for maximum growth-rate, were calculated using measurements of colony radius that were made only on the side of the colony adjacent to the potential biocontrol agent. Upon inoculation *P. infestans* 10LD3 and potential biological control agent were placed 26-mm apart. The maximum possible score for this parameter was 100%.

c. These values indicate maximum growth-rate within zone of mixed culture.

d. Inhibition coefficients were calculated by the following equation: (100 − growth-rate *B*) × 0.4 + (100 − growth *C*) × 0.4 + (100 − growth-rate *E*) × 0.2; see Cray *et al*., (2015); values which do not include the same letter(s) in brackets are significantly different (*P* < 0.05). Values in red indicate promotion of pathogen growth.

**Table S4.** Key parameters and inhibition coefficients, obtained from interaction assays between biocontrol agents and *Phytophthora infestans* 10D2_5^a^

| Potential biocontrol agents | *P. infestans* 10D2_5 growth rate (mm day^-1^); [*A*]^b^ | Growth rate [*A*] as a percentage of control; [*B*] | Growth of *P. infestans* 10D2_5 as a percentage of the distance between sites of inoculation; [*C*] | Time until contact between *P. infestans* 10D2_5 and biocontrol agent (days) | *P. infestans* 10D2_5 growth rate in zone of mixed culture (mm day^-1^); [D]^c^ | Growth rate [*D*] as percentage of control; [*E*] | Inhibition coefficient^d^ |
| --- | --- | --- | --- | --- | --- | --- | --- |
| *Bacillus subtilis* QST 713 | 0.97 ± 0.05 | 30.1 ± 1.50 | 12.5 ± 6.25 | 1.00 ± 0.00 | 0.00 ± 0.00 | 0.00 ± 0.00 | **83.0** ± 3.10(a) |
| JC12GB43 | 1.00 ± 1.00 | 30.9 ± 31.0 | 14.6 ± 13.0 | 2.15 ± 0.07 | 0.00 ± 0.00 | 0.00 ± 0.00 | **81.8** ± 17.6(a) |
| *Pseudomonas fluorescens* 2-79 | 1.38 ± 0.54 | 42.7 ± 16.7 | 14.6 ± 20.1 | 2.00 ± 0.00 | 0.00 ± 0.00 | 0.00 ± 0.00 | **77.1** ± 14.7(a) |
| JC12GB14 | 1.24 ± 0.52 | 38.5 ± 16.1 | 27.1 ± 9.56 | 7.96 ± 0.00 | 0.00 ± 0.00 | 0.00 ± 0.00 | **73.8** ± 10.3(ab) |
| JC12GB6 | 1.54 ± 0.53 | 47.6 ± 16.5 | 27.1 ± 13.0 | 4.88 ± 3.49 | 0.08 ± 0.14 | 2.50 ± 4.33 | **69.9** ± 12.7(abc) |
| JC12GB7 | 2.15 ± 1.07 | 66.6 ± 33.0 | 22.9 ± 9.56 | 5.54 ± 0.00 | 0.00 ± 0.00 | 0.00 ± 0.00 | **64.2** ± 17.0(abcd) |
| JC12GB13 | 1.85 ± 0.00 | 57.1 ± 0.00 | 37.5 ± 6.25 | No overlap between cultures | No overlap between cultures | 0.00 ± 0.00 | **62.2** ± 2.50(abcd) |
| *Pichia anomala* J121 | 1.96 ± 0.80 | 60.6 ± 24.6 | 35.4 ± 7.19 | No overlap between cultures | No overlap between cultures | 0.00 ± 0.00 | **61.6** ± 12.7(abcd) |
| *Streptomyces* sp. NC-1487 | 1.68 ± 0.59 | 52.0 ± 18.3 | 45.8 ± 9.56 | No overlap between cultures | No overlap between cultures | 0.00 ± 0.00 | **60.9** ± 11.1(abcd) |
| JC12GB29 | 2.06 ± 0.40 | 63.6 ± 12.3 | 41.7 ± 13.0 | No overlap between cultures | No overlap between cultures | 0.00 ± 0.00 | **57.9** ± 10.1(abcd) |
| JC12GB12 | 2.50 ± 0.57 | 77.3 ± 17.6 | 41.7 ± 3.63 | No overlap between cultures | No overlap between cultures | 0.00 ± 0.00 | **52.4** ± 8.47(abcd) |
| JC12GB34 | 3.10 ± 0.79 | 95.8 ± 24.5 | 43.8 ± 10.8 | No overlap between cultures | No overlap between cultures | 0.00 ± 0.00 | **44.2** ± 14.1(abcd) |
| *Pantoea* sp. S09:T:12 | 2.34 ± 0.52 | 72.3 ± 16.1 | 66.9 ± 28.9 | 5.00 ± 0.00 | 0.50 ± 0.87 | 15.5 ± 26.8 | **41.3** ± 23.3(abcd) |
| JC12GB50 | 2.90 ± 0.37 | 89.6 ± 11.6 | 75.0 ± 6.25 | No overlap between cultures | No overlap between cultures | 0.00 ± 0.00 | **34.2** ± 7.13(bcd) |
| JC12GB48 | 3.25 ± 0.32 | 100 ± 9.81 | 81.3 ± 6.25 | 5.08 ± 0.00 | 0.00 ± 0.00 | 0.00 ± 0.00 | **27.3** ± 6.43(cde) |
| JC12GB51 | 4.02 ± 0.67 | 124 ± 20.9 | 64.4 ± 3.63 | 5.96 ± 0.00 | 0.00 ± 0.00 | 0.00 ± 0.00 | **24.4** ± 9.79(de) |
| JC12GB47 | 4.02 ± 0.64 | 124 ± 19.9 | 70.6 ± 23.7 | 4.90 ± 2.68 | 0.00 ± 0.00 | 0.00 ± 0.00 | **22.0** ± 17.4(de) |
| JC12GB36 | 3.57 ± 0.61 | 111 ± 18.7 | 85.6 ± 3.63 | 4.54 ± 0.76 | 0.00 ± 0.00 | 0.00 ± 0.00 | **21.6** ± 8.94(de) |
| JC12GB35 | 3.68 ± 0.27 | 114 ± 8.47 | > 100 ± 0.00 | 4.25 ± 0.66 | 3.95 ± 2.36 | 122 ± 73.1 | **-9.94** ± 18.0(e) |
| JC12GB28 | 4.48 ± 0.55 | 139 ± 17.1 | > 100 ± 0.00 | 4.67 ± 0.58 | 6.83 ± 3.47 | 211 ± 107 | **-37.6** ± 28.3(f) |

a. All assays were conducted on CA and values are means of three independent replicates ± standard deviation.

b. These values, for maximum growth-rate, were calculated using measurements of colony radius that were made only on the side of the colony adjacent to the potential biocontrol agent. Upon inoculation *P. infestans* 10D2_5 and potential biological control agent were placed 16-mm apart.

c. These values indicate maximum growth-rate within zone of mixed culture.

d. Inhibition coefficients were calculated by the following equation: (100 − growth-rate *B*) × 0.4 + (100 − growth *C*) × 0.4 + (100 − growth-rate *E*) × 0.2; see Cray *et al*., (2015); values which do not include the same letter(s) in brackets are significantly different (*P* < 0.05). Values in red indicate promotion of pathogen growth.

**Table S5.** Key parameters and inhibition coefficients, obtained from interaction assays between *Bacillus* sp. JC12GB43 and *Fusarium coeruleum* and on PDA supplemented with either glycerol or urea.^a^

| Stressor added to medium (concentration) | | *F. coeruleum* growth rate (mm day^-1^); [*A*]^b^ | Growth rate [*A*] as a percentage of control; [*B*] | Growth of *F. coeruleum* as a percentage of the distance between sites of inoculation; [*C*] | Time until contact between *F. coeruleum* and biocontrol agent (days) | *F. coeruleum* growth rate in zone of mixed culture (mm day^-1^); [D]^c^ | Growth rate [*D*] as percentage of control; [*E*] | Inhibition coefficient^d^ |
| --- | --- | --- | --- | --- | --- | --- | --- | --- |
| No added solute | 1.33 ± 0.47 | 35.0 ± 12.3 | 71.7 ± 6.38 | not applicable  (no contact) | not applicable  (no contact) | 0.00 ± 0.00 | **57.3** ± 7.48(a) |  |
| Glycerol (1.84 M) | 1.68 ± 0.24 | 78.3 ± 11.2 | > 100 ± 0.00 | 12.6 ± 4.06 | 0.63 ± 0.38 | 29.6 ± 17.9 | **22.8** ± 6.61(b) |  |
| Glycerol (2.53 M) | 0.83 ± 0.01 | 82.6 ± 1.40 | > 100 ± 0.00 | 21.3 ± 2.72 | 0.62 ± 0.22 | 62.0 ± 21.9 | **14.6** ± 4.77(b) |  |
| Urea (68.4 mM) | 3.25 ± 0.29 | 110 ± 9.73 | > 100 ± 0.00 | 5.08 ± 0.00 | 0.65 ± 0.31 | 22.0 ± 10.6 | **11.6** ± 3.18(b) |  |
| Glycerol (2.17 M) | 1.14 ± 0.11 | 90.8 ± 8.94 | > 100 ± 0.00 | 18.2 ± 0.00 | 0.77 ± 0.50 | 60.9 ± 39.5 | **11.5** ± 5.89(b) |  |
| Urea (132 mM) | 2.77 ± 0.22 | 202 ± 16.2 | > 100 ± 0.00 | 6.49 ± 1.15 | 2.16 ± 1.35 | 158 ± 98.7 | **-52.5** ± 18.5(c) |  |
| Urea (255 mM) | 0.00 ± 0.00 | not applicable | 0.00 ± 0.00 | not applicable | not applicable | not applicable | not applicable |  |
| a. All values are means of three independent replicates ± standard deviation.  b. These values, for maximum growth-rate, were calculated using measurements of colony radius that were made only on the side of the colony adjacent to the potential biocontrol agent (see Fig. 8). Upon inoculation *F. coeruleum* and potential biological control agent were placed 16-mm apart.  c. These values indicate maximum growth-rate within zone of mixed culture.  d. Inhibition coefficients were calculated by the following equation: (100 − growth-rate *B*) × 0.4 + (100 − growth *C*) × 0.4 + (100 − growth-rate *E*) × 0.2; see Cray *et al*., (2015); values which do not include the same letter(s) in brackets are significantly different (*P* < 0.05). Values in red indicate promotion of pathogen growth. | | | | | | | |  |

**Table S6.** Colony development of *Fusarium coeruleum* on PDA supplemented with 132 mM urea in relation to exogenous addition of compatible solutes and other substances.

| Added solute | Concen-tration of solutions prior to addition (M) | Quantity of solutionadded (µl) | Total amount of solute added (mmol) | Kosmo-/chaotropic activity of added solution (J kg^-1^)^a^ | Average growth rate (mm d^-1^) | Radial growth rate of colony on the side proximal to the well (mm d^-1^) | Radial growth rate of colony on right-hand side (mm d^-1^)^b^ | Radial growth rate of colony on the side opposite to the well (mm d^-1^) | Radial growth rate of colony on left-hand side (mm d^-1^)^c^ |
| --- | --- | --- | --- | --- | --- | --- | --- | --- | --- |
| potassium phosphate dibasic | 1.4 | 200 | 0.28 | −3.864 | 3.228 ± 0.151 | 3.191 ± 0.165 | 3.189 ± 0.217 | 3.218 ± 0.176 | 3.314 ± 0.060 |
| trehalose | 1.5 | 200 | 0.3 | −3.180 | 2.776 ± 0.108 | 2.746 ± 0.114 | 2.779 ± 0.060 | 2.862 ± 0.055 | 2.718 ± 0.162 |
| proline | 6 | 200 | 1.2 | −6.912 | 2.620 ± 0.229 | 2.582 ± 0.215 | 2.651 ± 0.344 | 2.616 ± 0.249 | 2.630 ± 0.241 |
| fructose | 4 | 200 | 0.8 | 3.648 | 2.576 ± 0.219 | 2.497 ± 0.196 | 2.586 ± 0.234 | 2.690 ± 0.350 | 2.531 ± 0.125 |
| PEG 600 | 1.5 | 200 | 0.3 | −22.200 | 2.431 ± 0.259 | 2.326 ± 0.377 | 2.444 ± 0.265 | 2.466 ± 0.234 | 2.486 ± 0.282 |
| ethanol | 5.644 | 200 | 1.129 | 6.694 | 2.366 ± 0.360 | 2.324 ± 0.388 | 2.354 ± 0.408 | 2.402 ± 0.464 | 2.384 ± 0.416 |
| glycerol | 6.935 | 200 | 1.387 | 1.470 | 2.298 ± 0.262 | 2.142 ± 0.358 | 2.290 ± 0.258 | 2.323 ± 0.191 | 2.437 ± 0.284 |
| mannitol | 0.8 | 200 | 0.16 | −1.056 | 2.226 ± 0.259 | 2.182 ± 0.223 | 2.297 ± 0.308 | 2.276 ± 0.301 | 2.149 ± 0.334 |
| betaine | 4.5 | 200 | 0.9 | −22.950 | 2.173 ± 0.101 | 2.174 ± 0.094 | 2.159 ± 0.189 | 2.142 ± 0.060 | 2.216 ± 0.057 |
| ethyl acetate | 0.9 | 200 | 0.18 | 11.052 | 2.089 ± 0.122 | 2.054 ± 0.080 | 2.002 ± 0.090 | 2.079 ± 0.153 | 2.219 ± 0.070 |
| urea | 10 | 200 | 2 | 33.200 | 1.849 ± 0.340 | 1.730 ± 0.430 | 1.909 ± 0.306 | 1.846 ± 0.384 | 1.912 ± 0.420 |
| ammonium sulphate | 3.4 | 200 | 0.68 | −45.492 | 1.333 ± 0.227 | 1.289 ± 0.272 | 1.317 ± 0.270 | 1.373 ± 0.245 | 1.353 ± 0.263 |
| lithium chloride | 12 | 200 | 2.4 | 48.720 | 1.138 ± 0.250 | 1.018 ± 0.136 | 0.878 ± 0.173 | 1.324 ± 0.103 | 1.333 ± 0.232 |
| phenol | 0.7 | 200 | 0.14 | 20.200 | 0.333 ± 0.171 | 0.282 ± 0.254 | 0.348 ± 0.012 | 0.302 ± 0.280 | 0.398 ± 0.073 |

a. See Cray *et al.* (2013). As in earlier studies, chaotropic activity of substances is expressed as a positive value whereas kosmotropic activity is expressed using negative values (Hallsworth *et al*., 2003a; 2003b; 2007; Williams and Hallsworth, 2009; Bhaganna *et al*., 2010; Chin *et al*., 2010; Cray *et al*., 2013a; 2013b; 2015b; Bell *et al*., 2013; Rummel *et al*., 2014; Yakimov *et al*., 2015).

b. Radial growth-rate of the *F. coeruleum* colony on the right-hand side when looking from the colony towards the direction of the well.
c. Radial growth-rate of the *F. coeruleum* colony on the left-hand side when looking from the colony towards the direction of the well.

**Supporting references**

Bell, A.N.W., Magill, E., Hallsworth, J.E., and Timson, D.T. (2013) Effects of alcohols and compatible solutes on the activity of ß-galactosidase. *Appl Biochem Biotech* 169: 786–796.

Bhaganna, P., Volkers, R.J.M., Bell, A.N.W., Kluge, K., Timson, D.J., McGrath, J.W., *et al*. (2010) Hydrophobic substances induce water stress in microbial cells. *Microb Biotechnol* **3**: 701–716.

Chin, J.P., Megaw, J., Magill, C.L., Nowotarski, K., Williams, J.P., Bhaganna, P., *et al*. (2010) Solutes determine the temperature windows for microbial survival and growth. *Proc Natl Acad Sci USA* **107**: 7835–7840.

Cray, J.A., Russell, J.T., Timson, D.J., Singhal, R.S., and Hallsworth, J.E. (2013a) A universal measure of chaotropicity and kosmotropicity. *Environ Microbiol* **15**: 287–296.

Cray, J.A., Bell, A.N.W., Bhaganna, P., Mswaka, A.Y., Timson, D.J., and Hallsworth, J.E. (2013b) The biology of habitat dominance; can microbes behave as weeds? *Microb Biotechnol* **6**: 453–492.

Cray, J.A., Houghton, J.D., Cooke, L.R., and Hallsworth, J.E. (2015a) A simple inhibition coefficient for quantifying potency of biocontrol agents against plant-pathogenic fungi. *Biol Control* **81**: 93–100.

Cray, J.A., Stevenson, A., Ball, P., Bankar, S.B., Eleutherio, E.C.A., Ezeji, T.C. *et al*. (2015b) Chaotropicity: a key factor in product tolerance of biofuel-producing microorganisms. *Curr Opin Biotechnol* **33**: 228–259.

Hallsworth, J.E., Heim, S., and Timmis, K.N. (2003a) Chaotropic solutes cause water stress in *Pseudomonas putida*. *Environ Microbiol* **5**: 1270–1280.

Hallsworth, J.E., Prior, B.A., Nomura, Y., Iwahara, M., and Timmis, K.N. (2003b) Compatible solutes protect against chaotrope (ethanol)-induced, nonosmotic water stress. *Appl Environ Microbiol* **69**: 7032–7034.

Hallsworth, J.E., Yakimov, M.M., Golyshin, P.N., Gillion, J.L.M., D'Auria, G., Alves, F.L., *et al*. (2007) Limits of life in MgCl_2_-containing environments: chaotropicity defines the window. *Environ Microbiol* **9**: 803–813.

Rummel, J.D., Beaty, D.W., Jones, M.A., Bakermans, C., Barlow, N.G., Boston, P., *et al*. (2014) A new analysis of Mars ‘Special Regions’, findings of the second MEPAG Special Regions Science Analysis Group (SR-SAG2). *Astrobiology* **14**: 887–968.

Williams, J.P., and Hallsworth, J.E. (2009) Limits of life in hostile environments; no limits to biosphere function? *Environ Microbiol* **11**: 3292–3308.

Yakimov, M.M., Lo Cono, V., La Spada, G., Bortoluzzi, G., Messina, E., Smedile, F., *et al*. (2015) Microbial community of seawater-brine interface of the deep-sea brine Lake *Kryos* as revealed by recovery of mRNA are active below the chaotropicity limit of life. *Environ Microbiol* **17**: 364–382.
